# Supplementary material for: Purine and pyrimidine synthesis differently affect the strength of the inoculum effect for aminoglycoside and β-lactam antibiotics
Source: Microbiol Spectr. 2024 Oct 22;12(12):e01895-24. doi: 10.1128/spectrum.01895-24 (PMC11619438; doi:10.1128/spectrum.01895-24)
Supplement: Supplemental material — Fig. S1 to S20; Tables S1 to S10; Methods. [file spectrum.01895-24-s0001.pdf]

Supplemental material for "Purine and pyrimidine synthesis differently affect the strength of the inoculum effect for aminoglycoside and β-lactam antibiotics".

Daniella M. Hernandez et al.
Corresponding authors: rsmith@nova.edu

This PDF file includes:

Supplemental Methods
Supplemental Results
Figs. S1 to S20
Tables S1 to S10

Supplemental Methods

Antibiotics used and their concentrations

We used the following antibiotics in this study: kanamycin (Fisher Scientific), carbenicillin (Thermofisher), streptomycin (Fisher Scientific), and ciprofloxacin (Thermofisher). For experiments performed with *E. coli*, and except for ciprofloxacin, antibiotics were provided in 1 μg/mL increments. Ciprofloxacin was provided in 0.002 μg/mL increments. For *P. aeruginosa*, streptomycin was provided in 4 μg/mL increments, whereas carbenicillin was provided in 5 μg/mL increments.

pH experiments

E. coli was grown overnight. The following day, the bacteria were washed and inoculated at low initial density into a 96 well plate containing M9 medium with casamino acids (0.01%-1%) and with, or without, nitrogenous bases (5 mM adenine, 10 mM thymine, 10 mM cytosine, 10 mM uracil). We chose to use a low initial density of cells as it allows for the greatest period of growth before reaching stationary. Thus, any changes in pH would be more readily observable that those of a high-density counterpart. After 24 hours of growth (as described in 'MIC assays', main text), 100 μL of the medium was removed, and placed in 5 ml of dH_2O whereupon pH was recorded using a symphony B10P pH meter (VWR, Radnor, PA) that was calibrated right before measurements took place. Similarly, 100 μL of cell-free medium was placed in 5 mL of dH_2O, and initial pH was recorded.

RNA extractions

E. coli was grown overnight whereupon it was diluted 100-fold in 200 μL of fresh M9 medium in a 96 well plate. Two Breathe Easy sealing membranes were placed over top. After 5 hours of growth at 37°C (250RPM), cell density was recorded using $OD_{600,}$ and 200 μL of three technical replicates were combined in a 1.5 mL microcentrifuge tube. The cells were centrifuged for 2 minutes at 12,000 RPM and were then resuspended in 30 μL of lysozyme (10 mg/mL, MP Biomedicals). The tube was incubated at room temperature for 20 minutes with vortexing completed every 2 minutes. Total RNA was then extracted using a ZR Fungal/Bacterial RNA MiniPrep kit (Zymo Research) including the optional in-column DNase digest according to the manufacturer's recommendations. Total RNA was quantified using a Qubit fluorometer (ThermoFisher) using the broad range (BR) RNA quantification kit (ThermoFisher) and was normalized by cell density (OD_{600}).

Measuring growth rate

As performed previously (1), OD_{600} values were log-transformed and normalized to the initial minimum density, which removes artifacts and ensures that all growth data were initiated from the same starting point. Together, this helps to reduce the amount of error during curve fitting. Next, we used the logistic equation to determine the maximum growth rate over 10 hours of growth, which allowed most conditions to reach the stationary phase.

$$y = \frac{A}{\left\{1+\exp\left(\frac{4\mu m}{A}(\lambda - t)+2\right)\right\}} \qquad \text{(Eq. S1)}$$

$$y = A\exp\left\{-\exp\left(\left(\frac{\mu_m e}{A}\right)(\lambda - t) + 1\right)\right\} \quad \text{(Eq. S2)}$$

where A represents the maximum cell density, μ_m represents the maximal growth rate, and λ represents the lag time. Lower bounds of 0, and upper bounds of 2, 1, and 10 for A, μ_m, and λ respectively, were used. This step ensures that all parameters are within a biologically feasible parameter space. We then used MATLAB 2023b using the lsqcurvefit function, which is a non-linear least squares solver and serves to estimate values for A, μ_m, and λ that minimize the differences between the experimental data and fit. After fitting, we determined the average residual. Growth curves with residual values of 0.5 or greater were considered to lack a rigorous fit and were not included in our analysis. Owing to the challenge of fitting both fast (wildtype) and very slow (mutant) growing bacteria using a single model, this was relaxed in the case of the Δ*pyrC* and Δ*purK* mutants such that the mean average residual across all nitrogenous base concentrations was 0.5 or less.

Additional information on statistical analysis

We did not perform statistical analysis on ATPsyn, biomass, and additional flux values because FBA simulations are deterministic and only report a single set of values for a given parameter set. We did not perform a statistical analysis on [ATP]/growth rate values as both [ATP] and growth rate were measured from different biological replicates on different days. Therefore, they cannot be immediately paired to formulate a single [ATP]/growth rate value for each biological replicate that can be used for statistical analysis. Moreover, we could not find a well-established statistical analysis method that can account for the above while accounting for significant differences amongst [ATP] and growth rate values. Accordingly, we did not perform a statistical analysis on [ATP]/growth rate values throughout the manuscript. We do, however, test the significance of the error on [ATP]/growth relative to ΔMIC using Deming regressions, as noted throughout the manuscript.

Supplemental Results

Using a 0.01 cutoff for MIC experiments

After blanking OD_{600} for MIC assays, we set any condition that did not exceed a value of 0.01 to zero. This threshold was used to prevent fluctuations in OD_{600} values observed in cell-free medium counting as growth. Our previous work showed that OD_{600} of cell-free medium could fluctuate +/- 0.009. Thus, values below 0.01 are not robust indicators of bacterial growth. We showed previously that using this cutoff does not impact the general trends in ΔMIC (1).

Nitrogenous base import and downstream effects in E. coli

Previous research has demonstrated that both purines and pyrimidines provided in the growth medium can be imported into bacteria and affect nucleotide synthesis. For example, *E. coli* grown in minimal medium exogenously supplied with either 1 mM adenine (purine) or 1 mM uracil (pyrimidine) showed cytoplasmic accumulation of both nitrogenous bases using LC-MS/MS (2). Adenine can be imported into the cell using PurP (3), which can be converted to either AMP or inosine monophosphate (IMP) using purine nitrogenous base salvage pathways, both of which participate in purine biosynthesis. Accumulation of AMP reduces purine synthesis by repressing the activity of PurF. Cytosine can be imported into the cell using CodA/CodB (4), and is subsequently converted to uracil via CodA (5). Uracil is imported into the cell using UraA (6). In both cases, uracil is converted to UMP by Upp. UMP then participates in UTP and CTP *de novo* synthesis. Thymine can also be imported into bacteria using the *rut* pathway; RutG has been shown to import thymine at a rate of 0.3 nmol/mg, which is achieved after ~45 minutes of incubation (7). Upon import, thymine is converted to deoxythymidine by DeoA, which is subsequently converted to dTMP by Tdk (8, 9). *De novo* pyrimidine synthesis creates UMP, which through a series of reactions leads to the creation of dTMP by Tdk and ThyA (9). Accumulation of UMP inhibits additional pyrimidine synthesis by inhibiting CarA/CarB whereas the accumulation of dTTP inhibits Tdk activity (10). 5-phospho-α-D-ribose 1-diphosphate (prpp) serves as a link between *de novo* purine and pyrimidine synthesis as it is used as a substrate for both pathways, thus allowing both pathways to autoregulate (11). A reduction in activity in one pathway (e.g., purine synthesis) leads to the accumulation of prpp, which can subsequently be used for the other pathway (e.g., pyrimidine synthesis). Accordingly, and consistent with previous work (2), reducing the activity of pyrimidine synthesis through exogenously supplied pyrimidines increases purine synthesis. Similarly, reducing purine synthesis through exogenously supplied purines increases pyrimidine synthesis.

Import of IMP

Unlike other nitrogenous bases used in this study, IMP is not directly imported into the cells. Instead, IMP is imported into the periplasmic space using one of four different transporters (e.g., PhoE (12)). The 5'-nucleotidase UshA located in the periplasmic space likely converts IMP to inosine. Inosine is then imported into the cytosol by NupG (13). From here, Gsk in the adenine salvage pathway converts inosine to inosine monophosphate (14), which is then used for purine synthesis.

Alternative explanations for IE

ATP production: Previous work has shown that when the growth rate is held constant, increasing bacterial metabolism, as measured primarily through ATP production, is a better predictor of antibiotic lethality (15). While our previous work showed that growth productivity (Δ[ATP]/Δgrowth rate) is a better predictor of ΔMIC than log[ATP] alone (1), we wanted to test the relationship between log[ATP] and ΔMIC in the context of this study where exogenous nitrogenous bases are being provided in the growth medium. While we found significant relationships between log[ATP] and ΔMIC of streptomycin and carbenicillin for *E. coli*, the R^2 values (or the strength of the relationship) were lower than that of ΔMIC and log[ATP]/growth rate (Fig. S9). We did not find a significant relationship between log[ATP] and ΔMIC of ciprofloxacin. We also did not find a significant relationship between log[ATP] and ΔMIC of streptomycin and carbenicillin for *P. aeruginosa* (Fig. S11). Finally, a significant increase in [ATP] absent changes in growth rate relative to the no nitrogenous base control (1 mM adenine, Fig. S9) did not alter ΔMIC for streptomycin. Taken together, changes in [ATP] alone cannot fully explain IE.

Growth rate: Previous research has suggested that changes in growth rate alone can alter antibiotic lethality (16). While our previous work showed that growth rate alone cannot account for IE and changes in ΔMIC (1), we sought to test this in the context of this study. Linear regression analysis indicated that the relationship between growth rate and ΔMIC of streptomycin in *E. coli* was significant but less strong as compared to log[ATP]/growth rate (Fig. S9). We did not find a significant relationship between growth rate and ΔMIC of ciprofloxacin. Interestingly, we found a significant relationship between growth rate and ΔMIC of carbenicillin in *E. coli*, the strength of which was greater than that of ΔMIC of log[ATP]/growth rate. This may be owing to the strong dependence of growth rate on the efficacy of β-lactam antibiotics, which has been reported previously (17) and deserves future exploration. In addition, we found a moderately significant, but less strong, relationship between growth rate and ΔMIC of streptomycin for *P. aeruginosa* (Fig. S11). The same was not found when *P. aeruginosa* was challenged with carbenicillin. Finally, a significant reduction in growth rate absent changes in [ATP] relative to the no nitrogenous base control (0.9 mM thymine, Fig. S9) did not alter ΔMIC for streptomycin. Taken together, growth rate is not the most consistent predictor of ΔMIC when considering multiple bacterial species and antibiotics.

Carrying capacity: As the production of ATP is growth phase-dependent (18, 19), a higher carrying capacity in the growth medium may allow an extended log phase, which could impact antibiotic lethality and ΔMIC. To test the relationship between the carrying capacity of the growth medium (determined using the average OD_{600} reached by the high and low initial density populations in antibiotic-free medium), we performed a linear regression between ΔMIC and carrying capacity. For *E. coli*, we found significant relationships between carrying capacity and ΔMIC of streptomycin and carbenicillin (Fig. S10). However, the strength of these relationships was less than the relationships between ΔMIC and log[ATP]/growth rate. We did not find a significant relationship between carrying capacity and ΔMIC of ciprofloxacin. Taken together, carrying capacity alone is unlikely to be the strongest determinant of IE.

pH of the growth medium: Previous studies have shown that pH influences bacterial metabolism (20) and antibiotic lethality (21). As we did not use a buffered medium in our analysis, we wanted to ensure that alterations to pH due to bacterial growth or differences in media composition were not driving our results. Accordingly, we measured the pH of the medium both before and after

bacterial growth (see Supplemental Methods). The medium that was tested contained no nitrogenous bases, 5 mM adenine, 10 mM cytosine, 10 mM thymine, or 10 mM uracil. We averaged pH across the five percentages of casamino acids (0.01, 0.05, 0.1, 0.5, and 1%) and plotted the values against ΔMIC of streptomycin, carbenicillin, and ciprofloxacin. We did not find a significant nor strong relationship between ΔMIC and either initial or final pH (Fig. S10), which was consistent with our previous work (1).

Cell-cell communication: Previous work has indicated that secreted products produced by bacteria can confer antibiotic tolerance and resistance. These include molecules that create persister cells (22) and small molecules produced during quorum sensing (23). We previously showed that the removal of *tnaA*, which is implicated in the production of indole and the formation of persister cells (24), does not abolish IE (25). To test the effect of removing quorum sensing, we used a knockout strain that lacks *luxS*, which is involved in AI-2 production in *E. coli* (26). We found that when grown without nitrogenous bases or with 10 mM cytosine, IE continued to be present (Fig. S10). When grown with 10 mM thymine, and consistent with the wildtype strain containing *luxS*, ΔMIC was reduced relative to the no nitrogenous base control. Thus, quorum sensing does not appear to account for IE.

Antibiotic target to antibiotic ratio: Previous work has suggested that the ratio of antibiotic to antibiotic target can account for IE (27). For a given concentration of antibiotic, the greater the density of the population, the fewer antibiotic molecules per antibiotic target, which leads to increased tolerance. While our previous work provided evidence that this alternative explanation could not account for IE (1, 25), we explicitly tested this hypothesis in the context of our study by quantifying the total amount of RNA extracted from bacteria. Specifically, we wanted to ensure that the addition of nitrogenous bases was not reducing the concentration of ribosomes, thus increasing the ratio between antibiotic: antibiotic target, which could explain reductions in ΔMIC. Total RNA can be used as a reliable surrogate of the quantity of rRNA in the cell as 95–97% of total RNA is rRNA in bacteria (28) and tRNAs are removed during the purification process. We extracted total RNA from *E. coli* grown without exogenous nitrogenous bases, with 10 mM cytosine and with 10 mM thymine. We then correlated the quantity of total RNA normalized by cell density to ΔMIC of streptomycin; note that the molecular target of streptomycin is the ribosome. We did not find a significant nor strong relationship between the quantity of total RNA and ΔMIC of streptomycin (Fig. S10). This indicates that changes in the number of molecular targets as a result of exogenously applied nitrogenous bases cannot explain our findings.

Efflux pumps: Recent work has shown that the presence of efflux pumps can protect neighboring bacteria from the effects of antibiotics (29). Thus, it is possible that efflux pumps could account for density-dependent antibiotic resistance; conceivably, the higher the population density, the more efflux pumps would be available to collectively protect the population. To test this, we acquired a knockout strain that lacks the *mdtA* efflux pump, which confers antibiotic resistance (30). ΔMIC of this mutant was consistent when 10 mM cytosine was included in the medium as compared to a no nitrogenous base control (Fig. S10). Supplementation with 10 mM thymine reduced ΔMIC of the knockout strain as compared to the control. Our previous work has also shown that deletion of *tolC*, *acrA,* and *acrB*, all of which encode efflux pumps, continues to allow IE. Thus, efflux pumps are unlikely the leading cause of IE in our system (1).

ampC β-lactamase: *E. coli* strain BW25113 contains an endogenous *ampC* β-lactamase. However, carbenicillin is not readily broken down by the *ampC* β-lactamase (31). Moreover, our previous work showed that without the *ampC* β-lactamase (Δ*ampC*), IE remains present for carbenicillin in BW25113 (1). Taken together, AmpC is not playing a large role in determining IE for carbenicillin.

Antibiotic import due to changes in ATP: Previous work has shown that increasing ATP production by activating the TCA cycle can increase the import of aminoglycoside antibiotics. This increases their lethality (32). As we are increasing ATP production using nitrogenous bases, it is possible that increasing aminoglycoside import is potentiating the lethality of streptomycin. However, to our knowledge, ATP does not alter the import of β-lactams, such as carbenicillin. Moreover, the primary mode of action of β-lactams is to interrupt the function of penicillin-binding proteins, which are predominantly found extracellularly (33). Thus, it is unlikely that changes to the import of carbenicillin owing to an increase in ATP would impact its lethality.

Divalent cations: Previous work has shown that the amount of divalent cations in the growth medium can impact the ability of aminoglycosides to bind to the cell membrane prior to cell entry (34). However, as we do not vary the predominant sources of such cations (M9 salts, $MgSO_4$, $CaCl_2$) in our growth medium, this cannot explain our findings. Further to this, outside of their role in ensuring the functionality of some β-lactamases (35), we are unaware of literature indicating that the lethality of β-lactams is significantly impacted by divalent cations. Accordingly, changes in the concentration of divalent cations are unlikely driving our findings.

Flux balance analysis – parameter estimation

We performed both flux balance analysis and flux balance analysis with Optknock, using the COBRA toolbox v.3.0 (36) and the iML1515 model of *E. coli* (37). All lower and upper bound values that are considered standard in the iML1515 model were not altered. We altered the lower bound exchange values of K^+, Mg^{2+}, Na^+, NH_4^+, Cl^-, P_i, SO_4^{2-}, and Ca^{2+} to -1000 to account for the composition of M9 medium.

All lower bound exchange values were determined from previously published work and can be found in Table S1. All upper bound values remained 1000 except in the case where 6-MP was used in the medium (see *Flux balance analysis – simulations*). When determining g_{DW} from previous work, we assumed that 1 OD_{600} is approximately 0.39g/L cell dry weight (38). The lower bound exchange flux for amino acids was estimated as described previously (1) using published data (39). We approximated the order of magnitude of the lower bound flux value of glucose using previously published work (40, 41). The oxygen consumption rate (O2_tex) was estimated from previously published work (1, 42). Finally, the thiamine consumption rate was determined from previous literature (43).

To estimate the lower bound flux values of nitrogenous bases provided in the growth medium, we used previously published work to estimate the order of magnitude of uptake when ~ 1 μM of each nitrogenous base was separately provided to *E. coli* (3, 44-47). To account for experiments where the concentration of nitrogenous bases provided in the medium was 1-10 mM, we considered previous data showing that increasing the concentration of nitrogenous bases increases uptake near linearly (46) but can also saturate once higher concentrations (~0.8 mM) are reached (3). To take into account both trends, we first scaled the order of magnitude of the lower bound flux value linearly as a function of increasing concentration of nitrogenous bases. However, to account for the saturation of uptake at

high concentrations of nitrogenous bases, we decreased the lower bound value one order of magnitude from the linear scaling.

Flux balance analysis – simulations

We performed both flux balance analysis and flux balance analysis with Optknock, using the COBRA toolbox v.3.0 (36) and the iML1515 model of *E. coli* (37). MATLAB 2019b was used for simulations owing to its established compatibility with multiple optimization solvers. The LP solver of Gurobi was used as the optimization software (version 11.0.0). We used git version 2.39.2. For all simulations, biomass was set as the objective function.

For Optknock simulations, all 1506 genes were removed, and resulting changes in ATPsyn and biomass were evaluated. Biomass values of 0 were set to 10^{-14}, which was less than the lowest biomass value simulated (-1.26×10^{-13}). This was performed so that ATPsyn/biomass could be determined for genes whose removal was lethal (biomass = 0). Pathways, as identified using Ecocyc,(48) were then assigned to genes whose removal increased ATPsyn/biomass above that of the wildtype (simulations performed without knocking out any genes but using the same parameter set). We then calculated the frequency of the occurrence of each pathway and their average ATP/syn values (Fig. 1). Importantly, this was performed only for genes whose removal increased ATPsyn/biomass above wildtype.

For simulations that examined flux through purine and pyrimidine synthesis, we measured the flux of DHORTS and PRAIS (Fig. 7A). The DHORTS reaction captures the activity of the PyrC dihydroorotase. The PRAIS reaction captures the activity of the PurM phosphoribosylaminoimidazole synthase. Together, these reactions match the promoters of genes that were used for the reporter strain assays (*pyrC* and *purM* promoters). We note that simulation predictions with DHORTS and PRAIS are consistent when multiple reactions in pyrimidine (DHORTS, OMPDC, ORPT, AND ASPCT) and purine (IMPC, AIRC3, ADSL2R, AIRC2, GLUPRT, PRAGSR, PRASCSI, and PRAIS) synthesis are considered (Fig. S17).

For simulations with 6-MP (Fig. 7), we reduced the upper bound flux value of the GLUPRT reaction (captures the *purF* glutamine phosphoribosyldiphosphate amidotransferase reaction, which is inhibited by 6-MP (49)), which approximates the effect of an inhibitor by reducing the maximum flux through this part of purine synthesis. For simulations with IMP (Fig. 7), we modified the lower bound flux value of IMP exchange (EX_imp_e). As IMP uptake rates are not yet reported, we estimated the order of magnitude of this lower bound flux value using previously reported flux values of multiple carbon sources provided to *E. coli* (1). Sensitivity analysis for these parameters can be found in Fig. S17.

Supplemental References

1. Diaz-Tang G, Meneses EM, Patel K, Mirkin S, García-Diéguez L, Pajon C, Barraza I, Patel V, Ghali H, Tracey AP, Blanar CA, Lopatkin AJ, Smith RP. 2022. Growth productivity as a determinant of the inoculum effect for bactericidal antibiotics. Science Advances 8:eadd0924.
2. Yang JH, Wright SN, Hamblin M, McCloskey D, Alcantar MA, Schrübbers L, Lopatkin AJ, Satish S, Nili A, Palsson BO. 2019. A white-box machine learning approach for revealing antibiotic mechanisms of action. Cell 177:1649-1661. e9.
3. Burton K. 1994. Adenine transport in Escherichia coli. Proceedings of the Royal Society of London Series B: Biological Sciences 255:153-157.
4. Danielsen S, Kilstrup M, Barilla K, Jochimsen B, Neuhard J. 1992. Characterization of the Escherichia coli codBA operon encoding cytosine permease and cytosine deaminase. Molecular microbiology 6:1335-1344.
5. de Haan PG, Felix HS, Peters R. 1972. Mapping of the gene for cytosine deaminase on the Escherichia coli chromosome. Antonie Van Leeuwenhoek 38:257-63.
6. Lu F, Li S, Jiang Y, Jiang J, Fan H, Lu G, Deng D, Dang S, Zhang X, Wang J, Yan N. 2011. Structure and mechanism of the uracil transporter UraA. Nature 472:243-6.
7. Botou M, Lazou P, Papakostas K, Lambrinidis G, Evangelidis T, Mikros E, Frillingos S. 2018. Insight on specificity of uracil permeases of the NAT/NCS2 family from analysis of the transporter encoded in the pyrimidine utilization operon of Escherichia coli. Molecular Microbiology 108:204-219.
8. Kaminski PA. 2002. Functional Cloning, Heterologous Expression, and Purification of Two Different N-Deoxyribosyltransferases from Lactobacillus helveticus. Journal of Biological Chemistry 277:14400-14407.
9. Orchard SS, Goodrich-Blair H. 2005. Pyrimidine nucleoside salvage confers an advantage to Xenorhabdus nematophila in its host interactions. Applied and environmental microbiology 71:6254-6259.
10. Ke W, Saba JA, Yao C-H, Hilzendeger MA, Drangowska-Way A, Joshi C, Mony VK, Benjamin SB, Zhang S, Locasale J. 2020. Dietary serine-microbiota interaction enhances chemotherapeutic toxicity without altering drug conversion. Nature communications 11:2587.
11. Hove-Jensen B, Andersen KR, Kilstrup M, Martinussen J, Switzer RL, Willemoës M. 2017. Phosphoribosyl Diphosphate (PRPP): Biosynthesis, Enzymology, Utilization, and Metabolic Significance. Microbiol Mol Biol Rev 81.
12. Nikaido H. 2003. Molecular basis of bacterial outer membrane permeability revisited. Microbiology and molecular biology reviews 67:593-656.
13. Xie H, Patching SG, Gallagher MP, Litherland GJ, Brough AR, Venter H, Yao SY, Ng AM, Young JD, Herbert RB. 2004. Purification and properties of the Escherichia coli nucleoside transporter NupG, a paradigm for a major facilitator transporter sub-family. Molecular membrane biology 21:323-336.
14. Jochimsen B, Nygaard P, Vestergaard T. 1975. Location on the chromosome of Escherichia coli of genes governing purine metabolism: adenosine deaminase (add), guanosine kinase (gsk) and hypoxanthine phosphoribosyltransferase (hpt). Molecular and General Genetics MGG 143:85-91.
15. Lopatkin AJ, Stokes JM, Zheng EJ, Yang JH, Takahashi MK, You L, Collins JJ. 2019. Bacterial metabolic state more accurately predicts antibiotic lethality than growth rate. Nature microbiology:1-9.

16. Łapińska U, Voliotis M, Lee KK, Campey A, Stone MRL, Tuck B, Phetsang W, Zhang B, Tsaneva-Atanasova K, Blaskovich MA. 2022. Fast bacterial growth reduces antibiotic accumulation and efficacy. Elife 11:e74062.
17. Lee AJ, Wang S, Meredith HR, Zhuang B, Dai Z, You L. 2018. Robust, linear correlations between growth rates and β-lactam–mediated lysis rates. Proceedings of the National Academy of Sciences 115:4069-4074.
18. An JH, Goo E, Kim H, Seo Y-S, Hwang I. 2014. Bacterial quorum sensing and metabolic slowing in a cooperative population. Proceedings of the National Academy of Sciences 111:14912-14917.
19. Nyström T. 2004. Stationary-phase physiology. Annu Rev Microbiol 58:161-181.
20. Albert LS, Brown DG. 2015. Variation in bacterial ATP concentration during rapid changes in extracellular pH and implications for the activity of attached bacteria. Colloids and Surfaces B: Biointerfaces 132:111-116.
21. McArdle CD, Lagan KM, McDowell DA. 2018. Effects of pH on the Antibiotic Resistance of Bacteria Recovered from Diabetic Foot Ulcer Fluid: An In Vitro Study. Journal of the American Podiatric Medical Association 108:6-11.
22. Zarkan A, Matuszewska M, Trigg SB, Zhang M, Belgami D, Croft C, Liu J, El-Ouisi S, Greenhalgh J, Duboff JS. 2020. Inhibition of indole production increases the activity of quinolone antibiotics against E. coli persisters. Scientific reports 10:11742.
23. Rahmati S, Yang S, Davidson AL, Zechiedrich EL. 2002. Control of the AcrAB multidrug efflux pump by quorum-sensing regulator SdiA. Molecular microbiology 43:677-685.
24. Li G, Young KD. 2013. Indole production by the tryptophanase TnaA in Escherichia coli is determined by the amount of exogenous tryptophan. Microbiology 159:402-410.
25. Tan C, Smith R, Srimani J, Riccione K, Prasada S, Kuehn M, You L. 2012. The inoculum effect and band-pass bacterial response to periodic antibiotic treatment. Molecular Systems Biology 8:617.
26. Schauder S, Shokat K, Surette MG, Bassler BL. 2001. The LuxS family of bacterial autoinducers: biosynthesis of a novel quorum-sensing signal molecule. Molecular microbiology 41:463-476.
27. Udekwu KI, Parrish N, Ankomah P, Baquero F, Levin BR. 2009. Functional relationship between bacterial cell density and the efficacy of antibiotics. Journal of antimicrobial chemotherapy 63:745-757.
28. Rosenow C, Saxena RM, Durst M, Gingeras TR. 2001. Prokaryotic RNA preparation methods useful for high density array analysis: comparison of two approaches. Nucleic acids research 29:e112-e112.
29. Wen X, Langevin AM, Dunlop MJ. 2018. Antibiotic export by efflux pumps affects growth of neighboring bacteria. Scientific reports 8:15120.
30. Górecki K, McEvoy MM. 2020. Phylogenetic analysis reveals an ancient gene duplication as the origin of the MdtABC efflux pump. PloS one 15:e0228877.
31. Mazzariol A, Cornaglia G, Nikaido H. 2000. Contributions of the AmpC β-lactamase and the AcrAB multidrug efflux system in intrinsic resistance of Escherichia coli K-12 to β-lactams. Antimicrobial agents and chemotherapy 44:1387-1390.
32. Meylan S, Porter CB, Yang JH, Belenky P, Gutierrez A, Lobritz MA, Park J, Kim SH, Moskowitz SM, Collins JJ. 2017. Carbon sources tune antibiotic susceptibility in Pseudomonas aeruginosa via tricarboxylic acid cycle control. Cell chemical biology 24:195-206.
33. Barbas JA, Díaz J, Rodríguez-Tébar A, Vázquez D. 1986. Specific location of penicillin-binding proteins within the cell envelope of Escherichia coli. J Bacteriol 165:269-75.

34. Taber HW, Mueller JP, Miller PF, Arrow AS. 1987. Bacterial uptake of aminoglycoside antibiotics. Microbiological Reviews 51:439-457.
35. Bahr G, Gonzalez LJ, Vila AJ. 2021. Metallo-β-lactamases in the age of multidrug resistance: from structure and mechanism to evolution, dissemination, and inhibitor design. Chemical reviews 121:7957-8094.
36. Heirendt L, Arreckx S, Pfau T, Mendoza SN, Richelle A, Heinken A, Haraldsdóttir HS, Wachowiak J, Keating SM, Vlasov V. 2019. Creation and analysis of biochemical constraint-based models using the COBRA Toolbox v. 3.0. Nature protocols 14:639-702.
37. Monk JM, Lloyd CJ, Brunk E, Mih N, Sastry A, King Z, Takeuchi R, Nomura W, Zhang Z, Mori H. 2017. i ML1515, a knowledgebase that computes Escherichia coli traits. Nature biotechnology 35:904-908.
38. Glazyrina J, Materne E-M, Dreher T, Storm D, Junne S, Adams T, Greller G, Neubauer P. 2010. High cell density cultivation and recombinant protein production with Escherichia coli in a rocking-motion-type bioreactor. Microbial Cell Factories 9:1-11.
39. Zampieri M, Hörl M, Hotz F, Müller NF, Sauer U. 2019. Regulatory mechanisms underlying coordination of amino acid and glucose catabolism in Escherichia coli. Nature communications 10:1-13.
40. Orth JD, Thiele I, Palsson BØ. 2010. What is flux balance analysis? Nature biotechnology 28:245-248.
41. Jain R, Srivastava R. 2009. Metabolic investigation of host/pathogen interaction using MS2-infected Escherichia coli. BMC Systems Biology 3:1-11.
42. Andersen KB, von Meyenburg K. 1980. Are growth rates of Escherichia coli in batch cultures limited by respiration? Journal of Bacteriology 144:114-123.
43. Hollenbach AD, Dickson KA, Washabaugh MW. 2002. Thiamin transport in Escherichia coli: the mechanism of inhibition by the sulfhydryl-specific modifier N-ethylmaleimide. Biochimica et Biophysica Acta (BBA)-Biomembranes 1564:421-428.
44. Hatton CE, Brotherton DH, Spencer M, Cameron AD. 2022. Structure of cytosine transport protein CodB provides insight into nucleobase-cation symporter 1 mechanism. The EMBO Journal 41:e110527.
45. Roy-Burman S, Visser DW. 1975. Transport of purines and deoxyadenosine in Escherichia coli. Journal of Biological Chemistry 250:9270-9275.
46. Jensen KF, Leer JC, Nygaard P. 1973. Thymine Utilization in Escherichia coli K12 on the Role of Deoxyribose 1-Phosphate and Thymidine Phosphorylase. European Journal of Biochemistry 40:345-354.
47. Andersen PS, Frees D, Fast R, Mygind B. 1995. Uracil uptake in Escherichia coli K-12: isolation of uraA mutants and cloning of the gene. Journal of Bacteriology 177:2008-2013.
48. Karp PD, Paley S, Caspi R, Kothari A, Krummenacker M, Midford PE, Moore LR, Subhraveti P, Gama-Castro S, Tierrafria VH. 2023. The EcoCyc Database (2023). EcoSal Plus 11:eesp-0002-2023.
49. Zhang Y, Morar M, Ealick SE. 2008. Structural biology of the purine biosynthetic pathway. Cell Mol Life Sci 65:3699-724.
50. Baba T, Ara T, Hasegawa M, Takai Y, Okumura Y, Baba M, Datsenko KA, Tomita M, Wanner BL, Mori H. 2006. Construction of Escherichia coli K-12 in-frame, single-gene knockout mutants: the Keio collection. Molecular systems biology 2:2006.0008.
51. Wilson HR, Turnbough CL. 1990. Role of the purine repressor in the regulation of pyrimidine gene expression in Escherichia coli K-12. Journal of Bacteriology 172:3208-3213.

Supplemental Figures

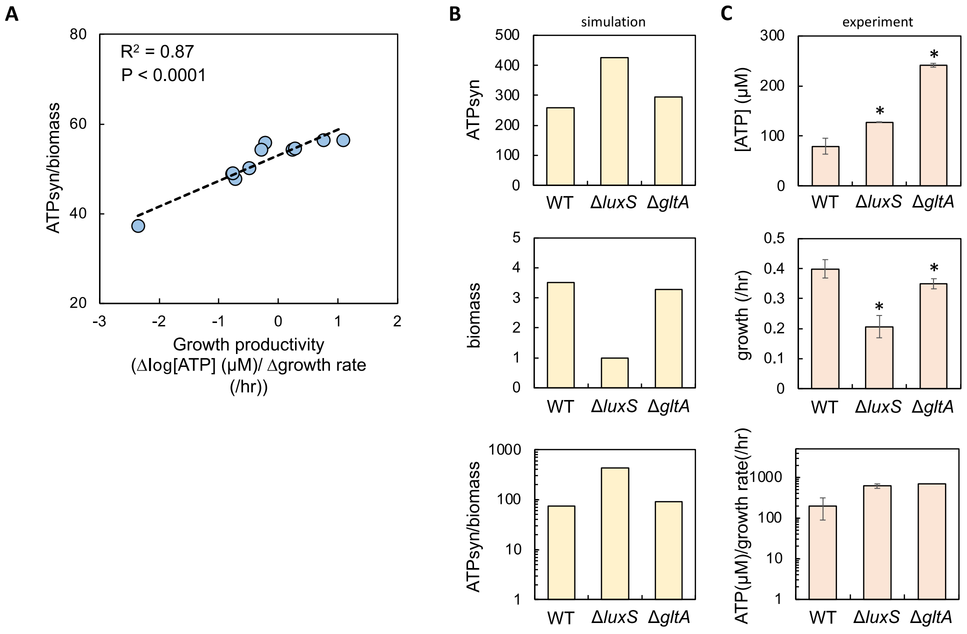

Fig. S1: Validation of flux balance analysis (FBA) and OptKnock to measure changes in [ATP] and growth rate. A) Relationship between flux balance analysis predicted ATPsyn/biomass values and experimentally determined values of growth productivity (change in ATP/change in growth rate) as defined previously (1). Data replotted from (1). P and R^2 from a linear regression. **B)** FBA + OptKnock predicted effects of removing *luxS* and *gltA* on ATPsyn, biomass, and ATPsyn/biomass. Parameters for FBA in Table S1. WT = wildtype. Note that Optknock predicts zero biomass production when removing *luxS*. To plot the data, we assigned a value of 1 to biomass for this simulation, which allows us to plot the data. **C)** Experimentally determined values of [ATP], growth rate, and [ATP]/growth rate of *luxS* and *gltA* knockouts as compared to WT. Δ*luxS* and Δ*gltA* were obtained from the Keio Collection (50). * = significantly different from WT ($P \leq 0.036$, one-tailed t-test). SEM from a minimum of three biological replicates. We note that while these knockouts match the trends predicted by FBA + OptKnock, this will not be the case for all knockouts as the current version of FBA does not contain all reactions in the cell.

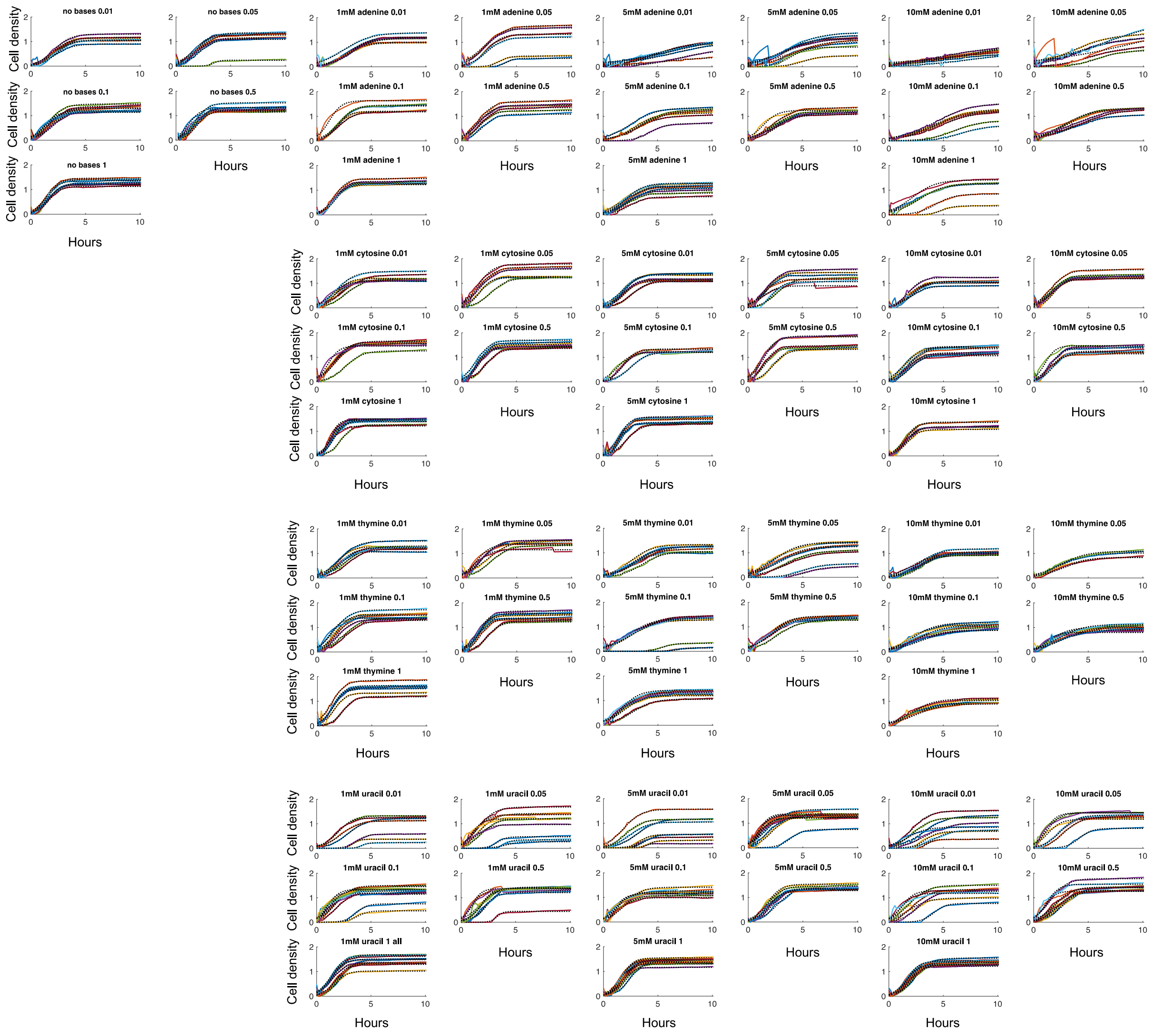

Fig. S2: Growth curves of *E. coli* grown in M9 medium with different concentrations of nitrogenous bases as indicated. The numbers at the top of each plot indicate the percentage of casamino acids (e.g., 0.01 = 0.01%). Colored lines = experimental data. Black dotted lines = growth curve fit using a logistic equation.

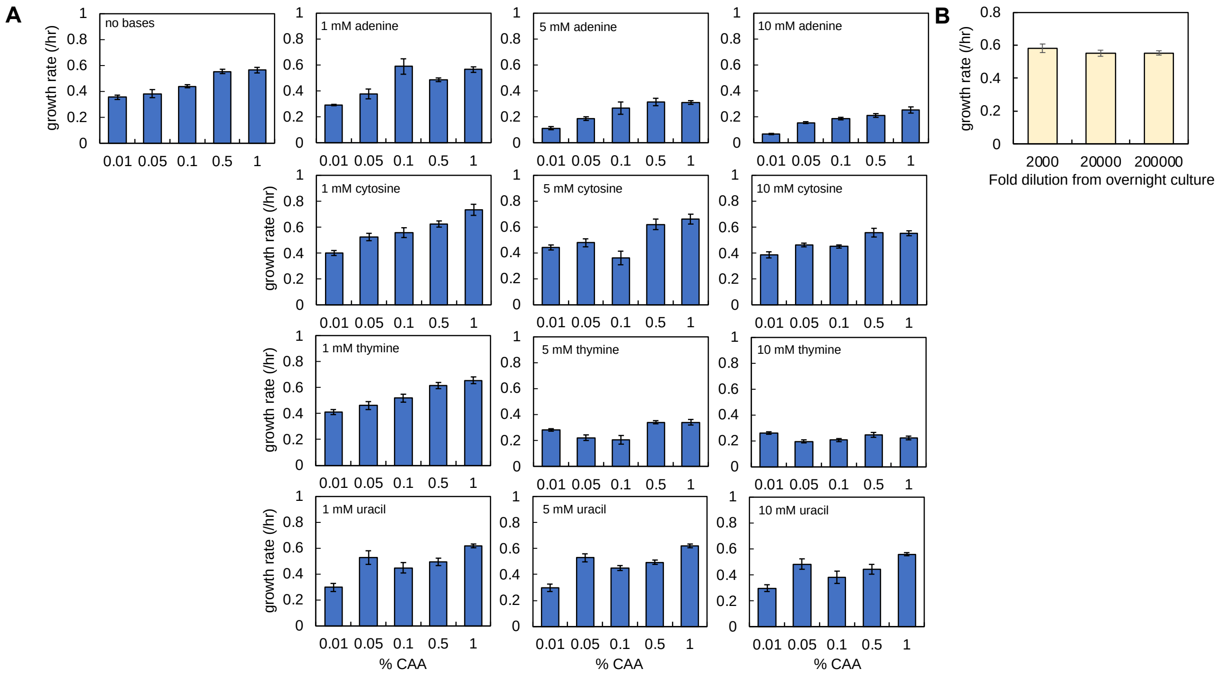

Fig. S3: Growth rates of *E. coli*. A) Raw growth rates for *E. coli* grown in M9 medium with different concentrations of nitrogenous bases as indicated on the plot. % CAA = percentage of casamino acids. Error bars = SEM. Bars = average from $\geq$ 4 biological replicates. **B)** The growth rate of *E. coli* at different initial densities covering the range of densities used in this study. We did not find significant differences in growth rate across these three densities. SEM from a minimum of four biological replicates.

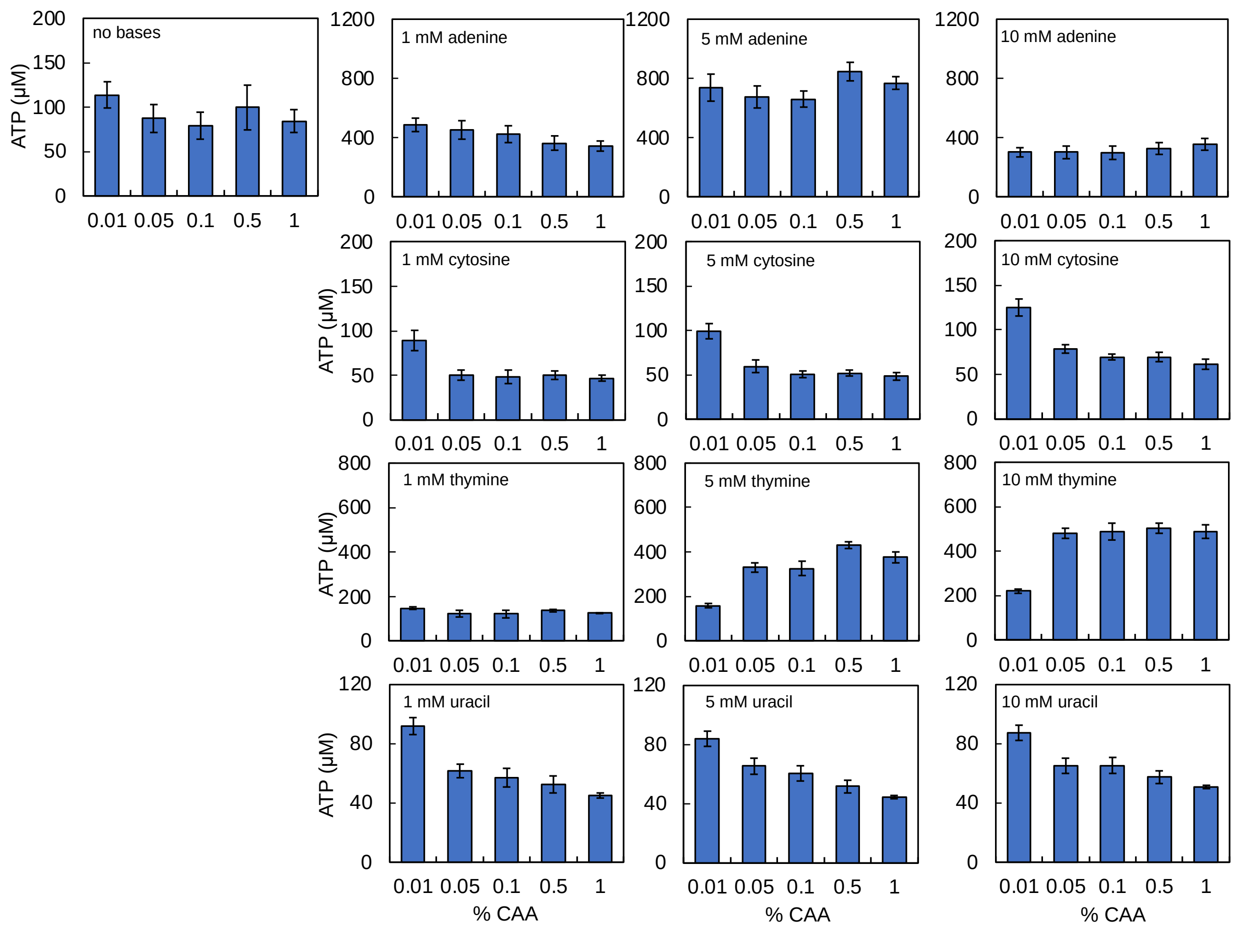

Fig. S4: Raw [ATP] for *E. coli* grown in M9 medium with different concentrations of nitrogenous bases as indicated. % CAA = percentage of casamino acids. Error bars = SEM. Bars = average from 4 biological replicates

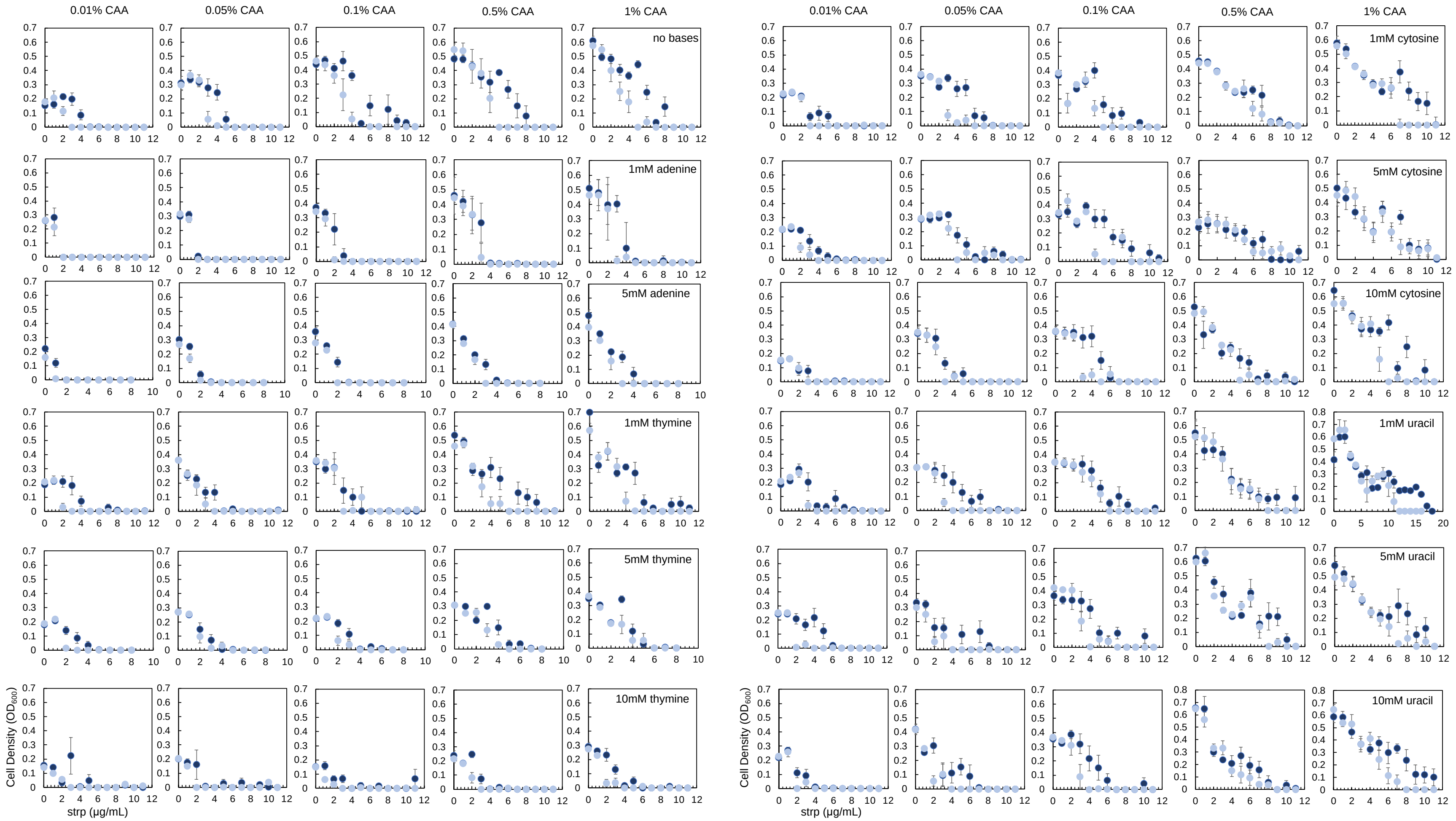

Fig. S5: Raw MIC data for *E. coli* grown in streptomycin (strp). % CAA = percentage of casamino acids. Error bars = SEM. Data corresponds to Fig. 3A. Each data point is the average of ≥ 5 biological replicates. Dark blue = high density; light blue = low density.

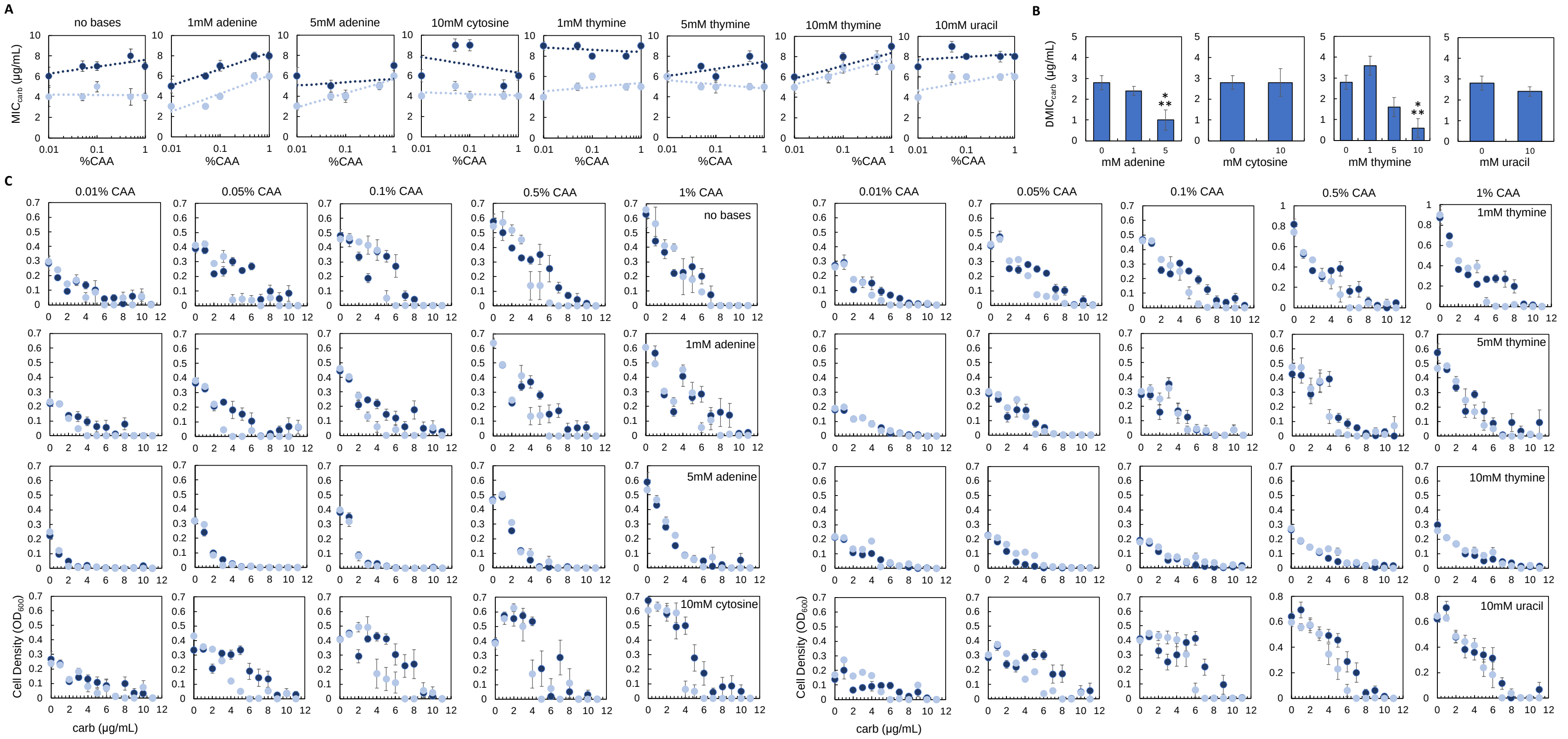

Fig. S6: MIC data for *E. coli* grown in carbenicillin.

A) MIC of carbenicillin (carb) as a function of the percentage of casamino acids. % CAA = percentage of casamino acids. Dark blue = high initial density, light blue = low initial density. For all panels error bars = SEM and each data point is the average of ≥ 5 biological replicates.

B) Average ΔMIC$_{carb}$ for each growth condition. Average plotted from 5 different % CAA each consisting of ≥ 5 biological replicates. Error bars = SEM. * different than no nitrogenous base control (P ≤ 0.03, two-tailed t-test); ** not different than zero (P ≥ 0.071, one-tailed t-test).

C) Raw data for panel A. Dark blue = high initial density, light blue = low initial density. Each data point is averaged from ≥ 5 biological replicates.

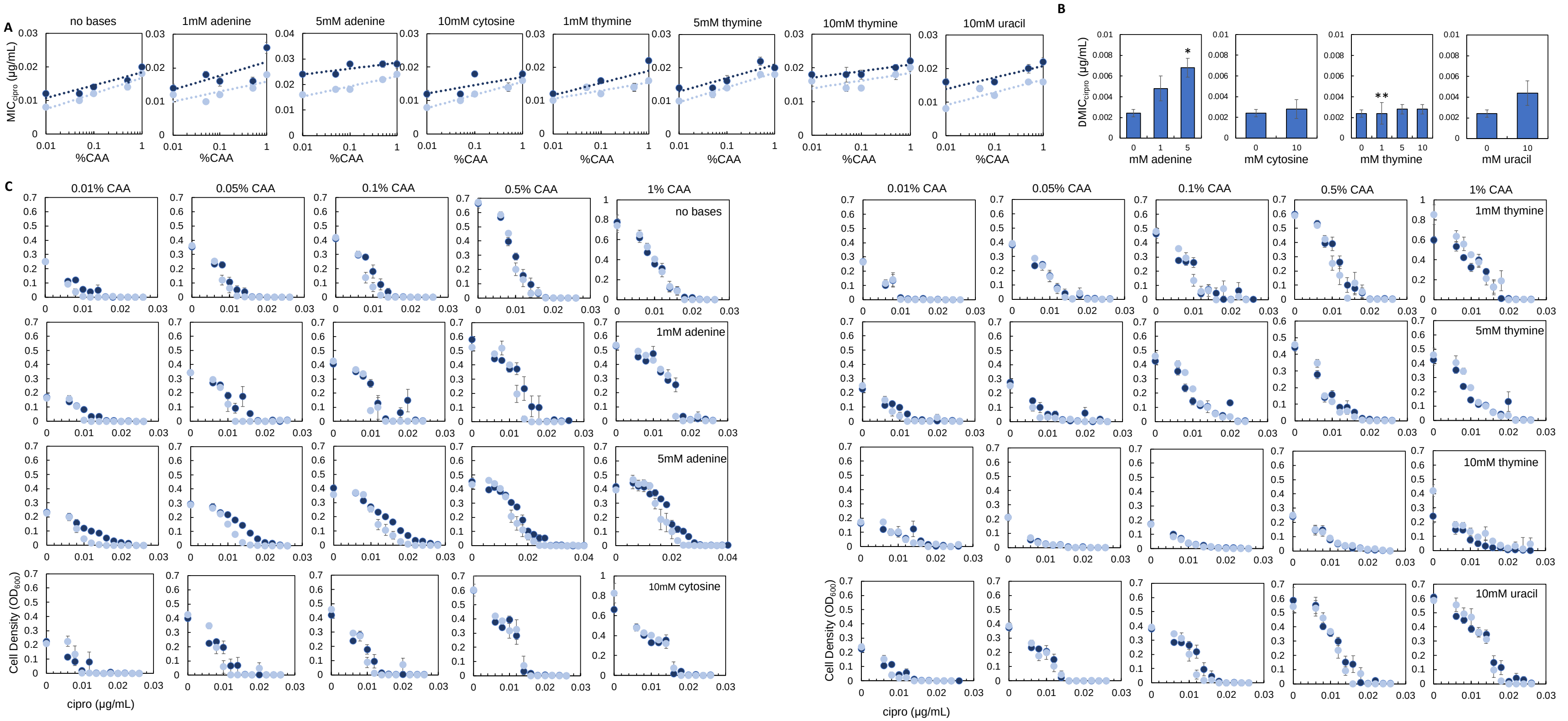

Fig. S7: MIC data for *E. coli* grown in ciprofloxacin.

A) MIC of ciprofloxacin (cipro) as a function of the percentage of casamino acids. % CAA = percentage of casamino acids. Dark blue = high initial density, light blue = low initial density. For all panels error bars = SEM and each data point is the average of ≥ 5 biological replicates.

B) Average ΔMIC_{cipro} for each growth condition. Average plotted from 5 different % CAA each consisting of ≥ 5 biological replicates. Error bars = SEM. * different than no nitrogenous base control (P = 0.009, two-tailed t-test); ** not different than zero (P = 0.054, one-tailed t-test).

C) Raw data for panel A. Dark blue = high initial density, light blue = low initial density. Averages plotted from ≥ 5 biological replicates.

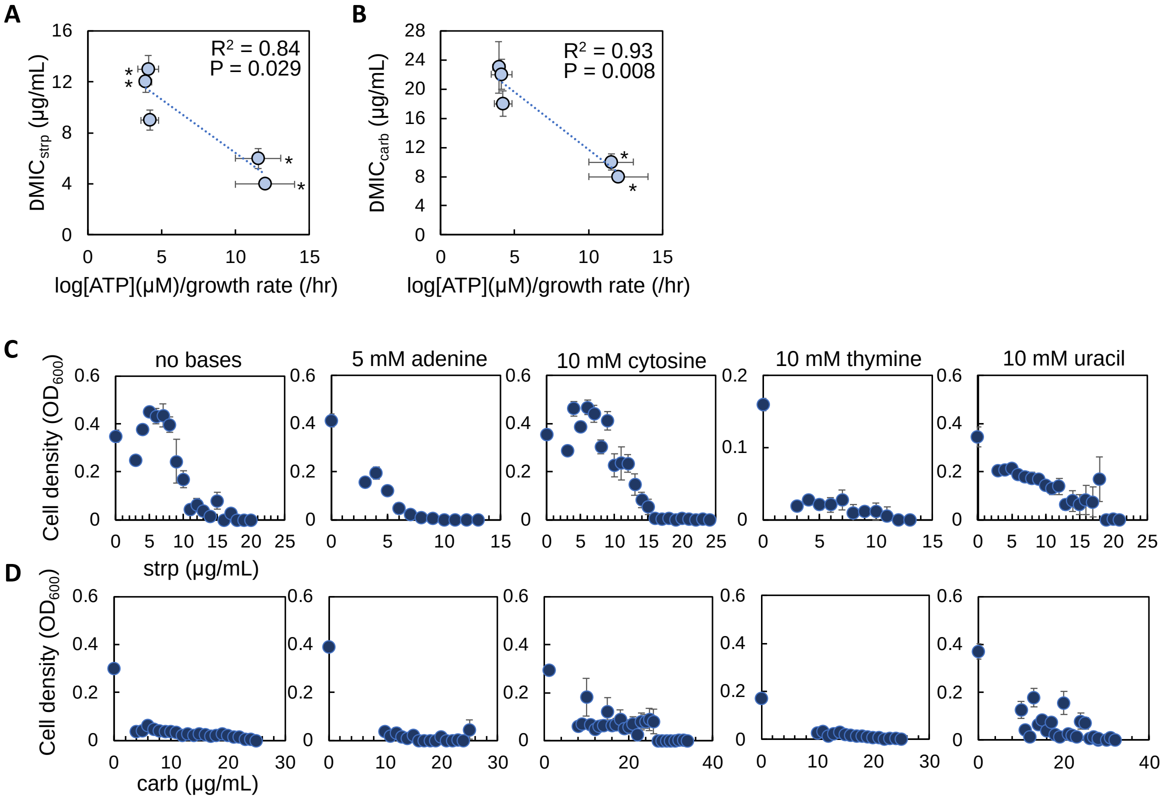

Fig. S8: The relationship between log[ATP]/growth rate and ΔMIC when a higher density of bacteria is used (2.00 x 10^7 CFU/mL +/- 4.50 x 10^6).

- **A)** ΔMIC of streptomycin (strp) as a function of log[ATP]/growth rate. R^2 and P value from a linear regression.* significantly different than no nitrogenous base control ($P \leq 0.002$, two-tailed t-test). log[ATP]/growth from Fig. 2C. Average plotted from ≥ 6 biological replicates. Error bars = SEM. WLS: $R^2 = 0.89$, $P = 0.015$, Deming regression: $P = 0.0290$. For all panels, ΔMIC was only measured using medium with 0.1% CAA.
- **B)** ΔMIC of carbenicillin (carb) as a function of log[ATP]/growth rate. R^2 and P value from a linear regression.* significantly different than no nitrogenous base control ($P \leq 0.001$, two-tailed t-test); log[ATP]/growth from Fig. 2C. Average plotted from ≥ 4 biological replicates. Error bars = SEM. WLS: $R^2 = 0.83$, $P = 0.032$, Deming regression: $P = 0.033$.
- **C)** Raw MIC data for experiments with a higher density of bacteria challenged with strp. Average from ≥ 4 biological replicates. Error bars = SEM.
- **D)** Raw MIC data for experiments with a higher density of bacteria challenged with carb. Average from ≥ 4 biological replicates. Error bars = SEM.

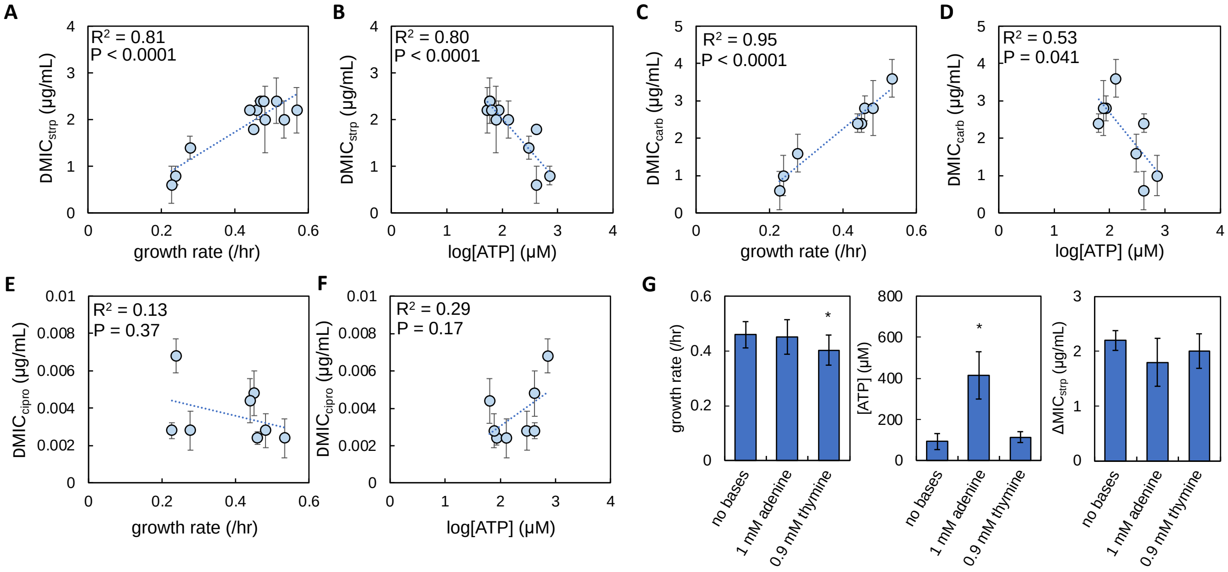

Fig. S9: Regression analysis between ΔMIC, and growth rate, log[ATP], and carrying capacity for each antibiotic.

- **A)** The relationship between ΔMIC of streptomycin (strp) and growth rate (/hr). For panels A-F, data from Fig. 2 (growth and [ATP]) and 3 (ΔMIC). R^2 and P value from a linear regression. Error bars = SEM.
- **B)** The relationship between ΔMIC of strp and log[ATP].
- **C)** The relationship between ΔMIC of carbenicillin (carb) and growth rate.
- **D)** The relationship between ΔMIC of carb and log[ATP].
- **E)** The relationship between ΔMIC of ciprofloxacin (cipro) and growth rate.
- **F)** The relationship between ΔMIC of cipro and log[ATP].
- **G)** The effect of changing only growth rate or [ATP] using nitrogenous bases on the ΔMIC of strp. Errors = SEM. Average [ATP] from four biological replicates. Average growth rate from ≥ 6 biological replicates. ΔMIC from ≥ 4 biological replicates. * = statistically different from no nitrogenous base control. Statistics using two-tailed t-test: growth rate (1 mM adenine vs control, $P = 0.567$; 0.9 mM thymine vs control, $P = 0.031$), [ATP] (1 mM adenine vs control, $P < 0.001$; 0.9 mM thymine vs control, $P = 0.086$), ΔMIC (1 mM adenine vs control, $P = 0.48$; 0.9 mM thymine vs control, $P = 0.61$).

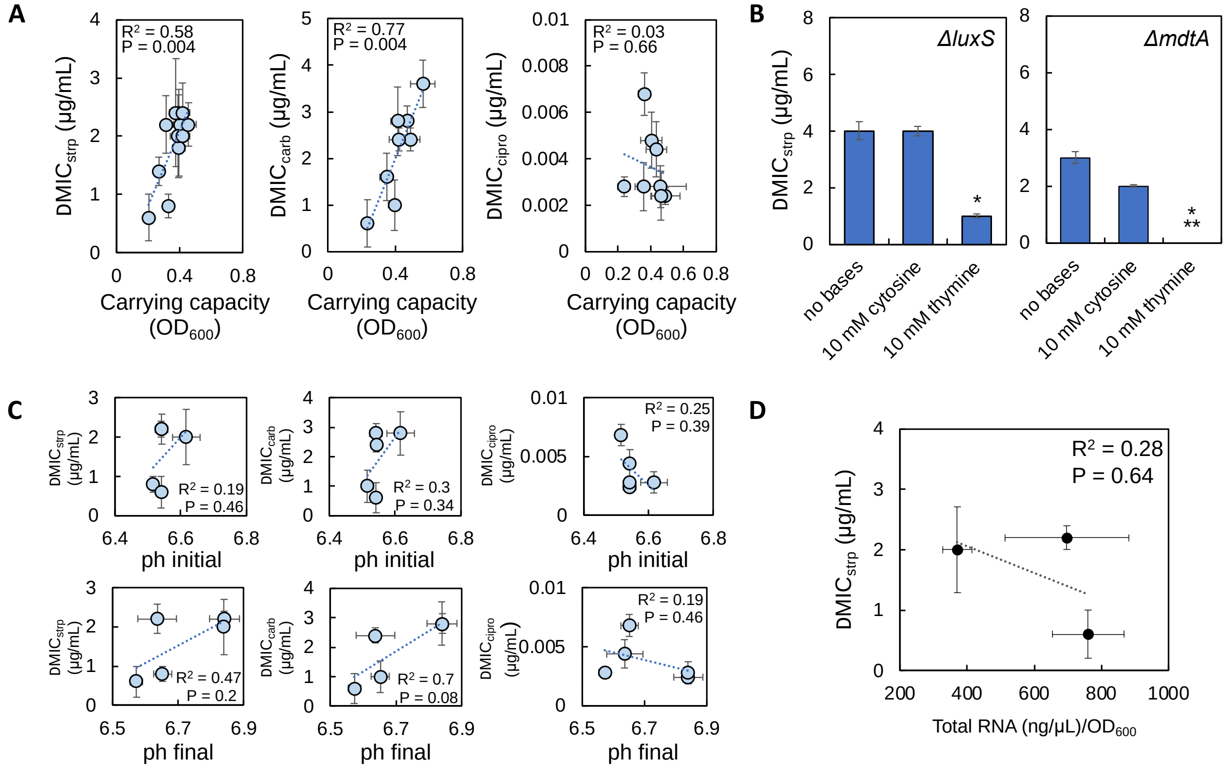

Fig. S10: Alternative explanations for IE.

A) The relationship between ΔMIC of strp (left), carb (center), and cipro (right) and carrying capacity of the growth medium (measured using OD_{600} of bacteria grown in the absence of antibiotics; averaged from both high and low density populations).

B) ΔMIC of streptomycin (strp) for *E. coli* strains that lack quorum sensing (*luxS*) or efflux pumps (*mdtA*). All strains retained IE and supplementation with 10 mM thymine, but not 10 mM cytosine, reduced ΔMIC. Error bars = SEM. Average from ≥ 5 biological replicates. * indicates less than no nitrogenous base (no bases.) control (P ≤ 0.019, two-tailed t-test). ** indicates not different than zero (P = 0.104, one-tailed t-test).

C) Linear regression between the pH of the growth medium before (top) and after (bottom) 24 hours of bacterial growth. The medium does not contain antibiotics. Error bars = SEM. pH averaged from 3 biological replicates. ΔMIC from Fig. 3.

D) The effect of adding 10 mM cytosine or 10 mM thymine on the concentration of rRNA in the cell. Error bars = SEM. Average from ≥ 2 biological replicates consisting of 3 technical replicates. ΔMIC of strp from Fig. 3. P = 0.15, ANOVA (P > 0.16, Tukey's HSD all comparisons).

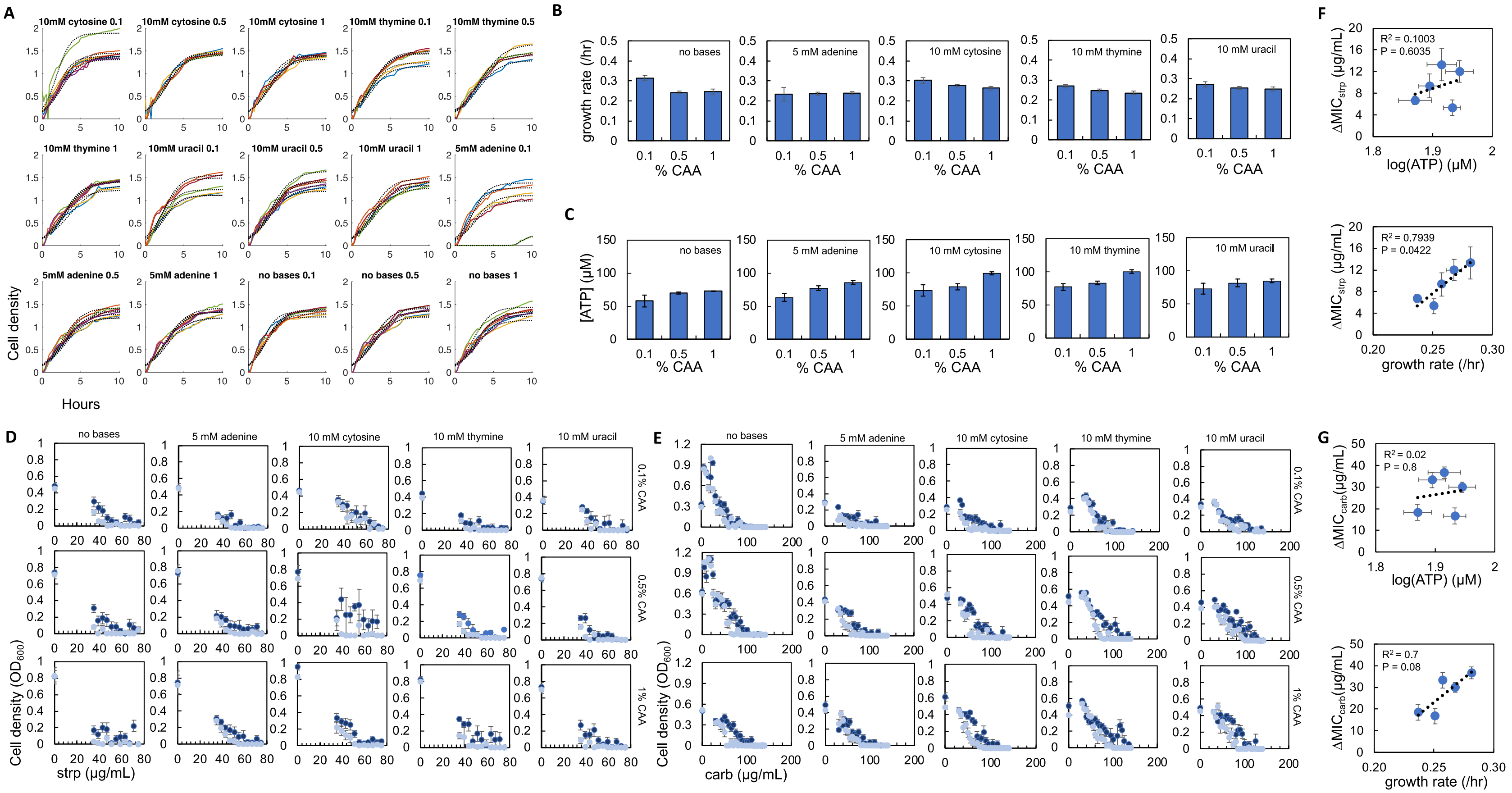

Fig. S11: Raw data for *P. aeruginosa*.

- **A)** Growth curves of *P. aeruginosa* grown in M9 medium with different concentrations of nitrogenous bases as indicated**.** The numbers at the top of each plot indicate the percentage of casamino acids (0.1 = 0.1% CAA). Colored lines = experimental data. Black dotted lines = growth curve fit using logistic equation.
- **B)** Average growth rate for each percentage of casamino acids (CAA) used. Error bars = SEM. Average plotted from ≥ 3 biological replicates.
- **C)** Average [ATP] for each % CAA and with, or without, nitrogenous bases as indicated. Error bars = SEM. Average plotted from ≥ 4 biological replicates.

D) Raw MIC data for *P. aeruginosa* grown in streptomycin (strp). Error bars = SEM. Average from ≥ 5 biological replicates. Dark blue = high density; light blue = low density.

E) Raw MIC data for *P. aeruginosa* grown in carbenicillin (carb). Error bars = SEM. Average from ≥ 5 biological replicates. Dark blue = high density; light blue = low density.

F) The relationship between ΔMIC of strp, [ATP] (top panel), and growth rate (bottom panel). ΔMIC, growth rate, and [ATP] from Fig. 4. R^2 and P value from a linear regression. Error bars = SEM.

G) The relationship between ΔMIC of carb, [ATP] (top panel), and growth rate (bottom panel). ΔMIC, growth rate, and [ATP] from Fig. 4. R^2 and P value from a linear regression. Error bars = SEM.

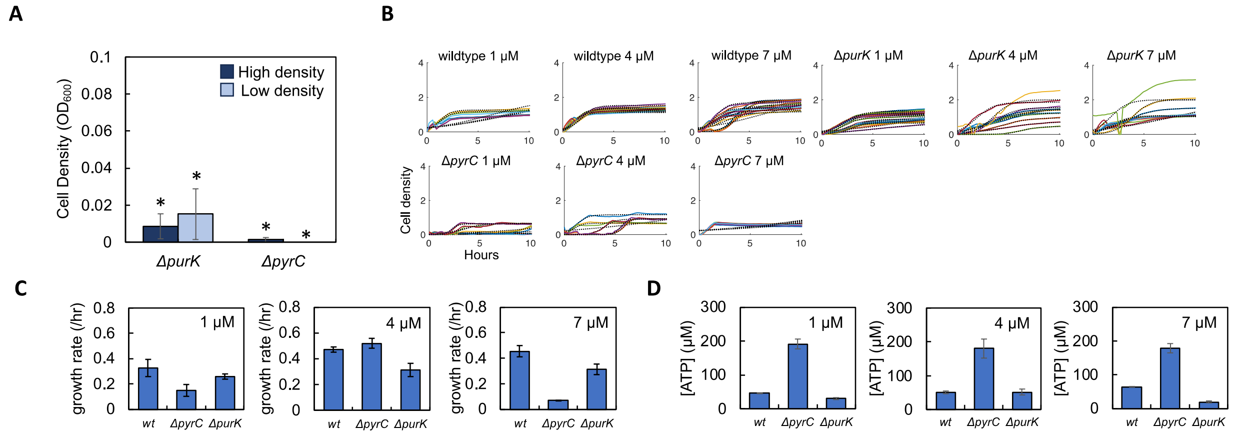

Fig. S12: Raw data for growth, growth rates, and [ATP] for knockout strains.

- **A)** Cell density (OD_{600}) of Δ*pyrC* and Δ*purK* knockout strains grown in M9 medium without exogenously supplemented nitrogenous bases. Average from three biological replicates of bacteria in medium with 0.01%, 0.05%, 0.1%, 0.5% and 1% casamino acids. Error bars = SEM. $P > 0.16$ when compared against zero (* = not different than zero, one-tailed t-test).
- **B)** Growth curves of *E. coli* knockout strains grown in M9 medium with different concentrations of equimolar nitrogenous bases as indicated in the plot. Colored lines = experimental data. Black dotted lines = growth curve fit.
- **C)** Raw growth rates. Concentration of equimolar nitrogenous bases is indicated on the plot. Error bars = SEM. Bars = average from ≥ 5 biological replicates.
- **D)** Raw [ATP] data. Concentration of equimolar nitrogenous bases is indicated on the plot. Error bars = SEM. Average from 3 biological replicates.

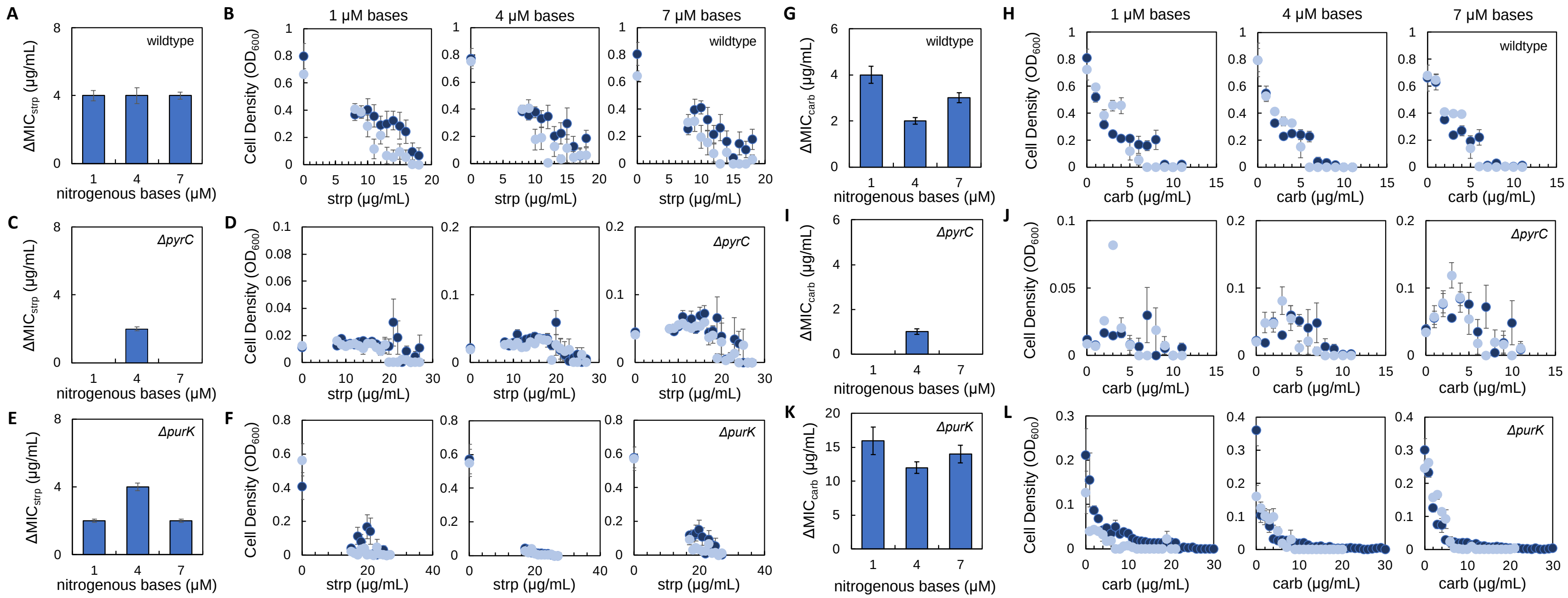

Fig. S13: Raw MIC data for purine and pyrimidine synthesis knockout strains.

- **A)** ΔMIC of streptomycin (strp) for wildtype *E. coli* as a function of the concentration of equimolar nitrogenous bases provided in the growth medium. Average from ≥ 5 biological replicates. For all panels, error bars = SEM.
- **B)** Raw data for panel A. For panels B, D, F, H, J, and L, dark blue = high initial density, light blue = low initial density.
- **C)** ΔMIC of strp for Δ*pyrC* as a function of the concentration of equimolar nitrogenous bases provided in the growth medium. Average from ≥ 5 biological replicates.
- **D)** Raw data for panel C.
- **E)** ΔMIC of strp for Δ*purK* as a function of the concentration of equimolar nitrogenous bases provided in the growth medium. Average from ≥ 5 biological replicates.
- **F)** Raw data for panel E.
- **G)** ΔMIC of carbenicillin (carb) for wildtype *E. coli* as a function of the concentration of equimolar nitrogenous bases provided in the growth medium. Average from ≥ 5 biological replicates.

H) Raw data for panel G.

I) ΔMIC of carb for Δ*pyrC* as a function of the concentration of equimolar nitrogenous bases provided in the growth medium. Average from ≥ 5 biological replicates.

J) Raw data for panel I.

K) ΔMIC of carb for Δ*purK* as a function of c equimolar nitrogenous bases provided in the growth medium. Average from ≥ 5 biological replicates.

L) Raw data for panel K.

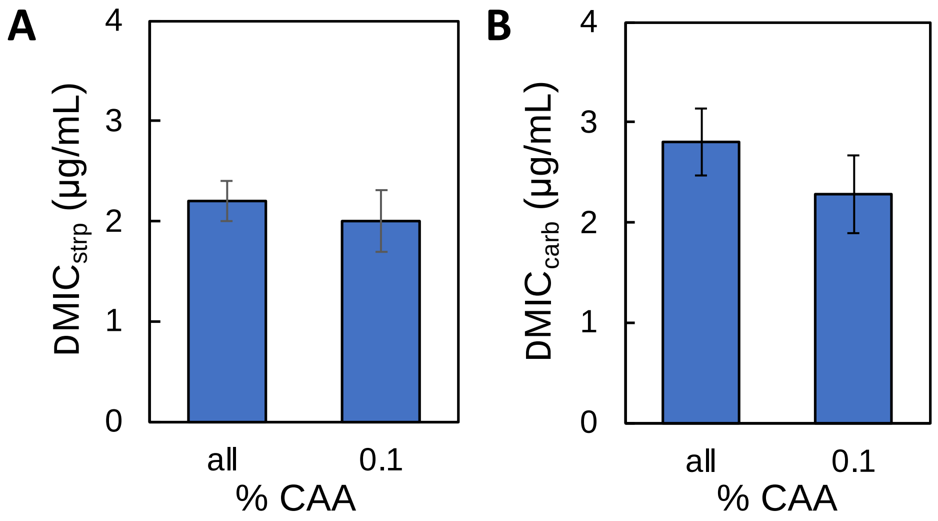

Fig. S14: Control data for Figure 6. ΔMIC of streptomycin (strp, panel A), and carbenicillin (carb, panel B) over the range of casamino acids (% CAA, 0.01%-1%) tested and using only 0.1% casamino acids. For each antibiotic when ΔMIC using all % CAA is compared to ΔMIC using 0.1%, $P \geq 0.38$, two-tailed t-test.

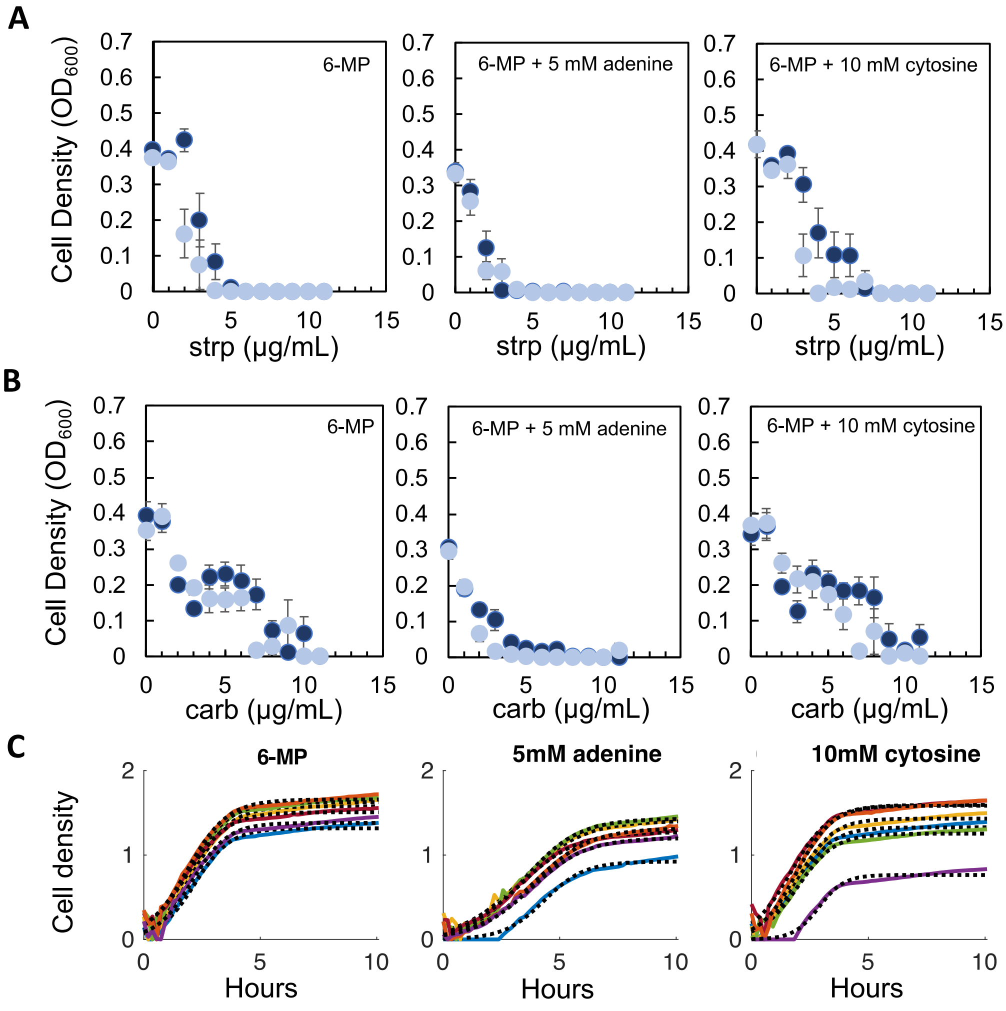

Fig. S15: Raw data for experiments using 6-MP.

A) Raw data from which the ΔMIC of streptomycin (strp) was determined when bacteria were challenged with 6-MP. Dark blue = high density, light blue = low density. Error bars = SEM. Average from ≥ 6 biological replicates.

B) Raw data from which the ΔMIC of carbenicillin (carb) was determined when bacteria were challenged with 6-MP. Dark blue = high density, light blue = low density. Error bars = SEM. Average from ≥ 7 biological replicates.

C) Growth curves of bacteria treated with 6-MP. Colored lines = experimental data. Black dotted lines = growth curve fit using logistic equation.

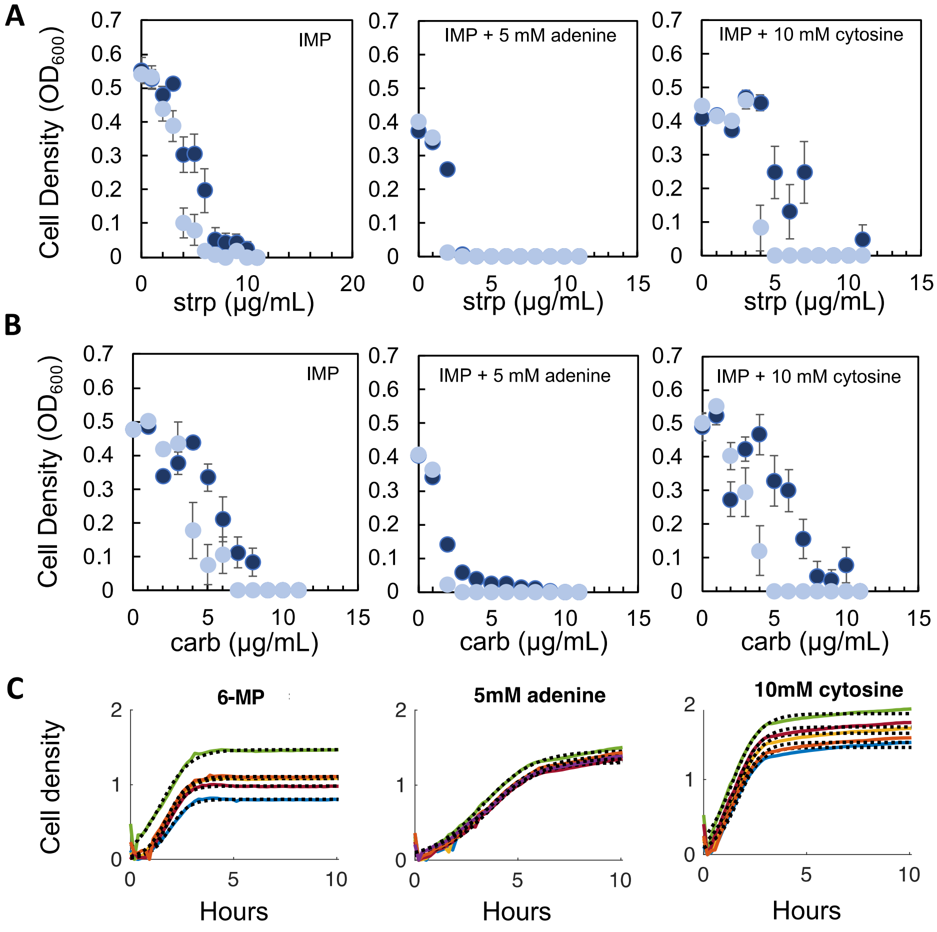

Fig. S16: Raw data for experiments using IMP.

A) Raw data from which the ΔMIC of streptomycin (strp) was determined when bacteria were challenged with IMP. Dark blue = high density, light blue = low density. Error bars = SEM. Average from ≥ 6 biological replicates

B) Raw data from which the ΔMIC of carbenicillin (carb) was determined when bacteria were challenged with IMP. Dark blue = high density, light blue = low density. Error bars = SEM. Average from ≥ 5 biological replicates.

C) Growth curves of bacteria treated with IMP. Colored lines = experimental data. Black dotted lines = growth curve fit using logistic equation.

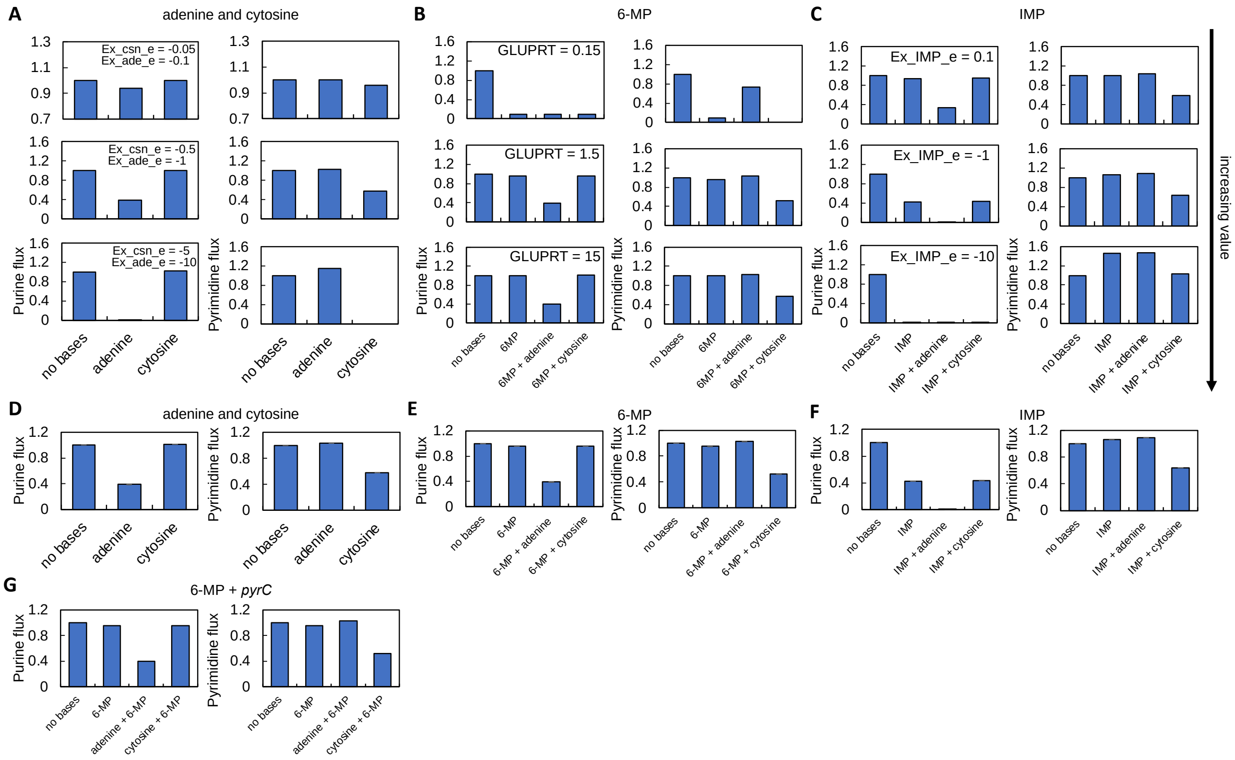

Fig. S17: Sensitivity analysis and additional FBA predictions.

- **A)** The effect of changing the lower bound exchange values of cytosine and adenine. Lower bound values are indicated on the plot. For panels A-C, the baseline simulation used for the main text is shown in the middle row. Purine flux represents the reaction catalyzed by PyrC (DHORTS); purine flux represents the reaction catalyzed by PurK (PRAIS).
- **B)** The effect of changing the upper bound value of the GLUPRT reaction, which affects the activity of the PurF enzyme. PurF is the target of 6-MP.
- **C)** The effect of changing the lower bond value of IMP exchange (Ex_IMP_e).
- **D-F)** Average flux values for all reactions involved in nucleotide synthesis up until the synthesis of IMP (purines, after which the pathway branches to produce AMP and GMP) and UMP (pyrimidine). SEM from all reactions. For panels D-F, reactions measured include DHORTS, OMPDC, ORPT, and ASPCT for pyrimidine flux, and IMPC, AIRC3, ADSL2R, AIRC2, GLUPRT, PRAGSR, PRASCSI, and PRAIS for purine flux.
- **G)** FBA predicted changes in purine (PRAIS) and pyrimidine (DHORTS) synthesis when 6-MP affects both *purF* (GLUPRT) and *pyrC* (DHORTS). These simulations were performed as a study found that PurR, a regulator of purine synthesis and a potential target of 6-MP, also controls the expression of *pyrC* (51). These simulations are consistent with simulations showing the impact of inhibiting PurF alone (Figure 7). The upper bound of DHORTS = 1.5.

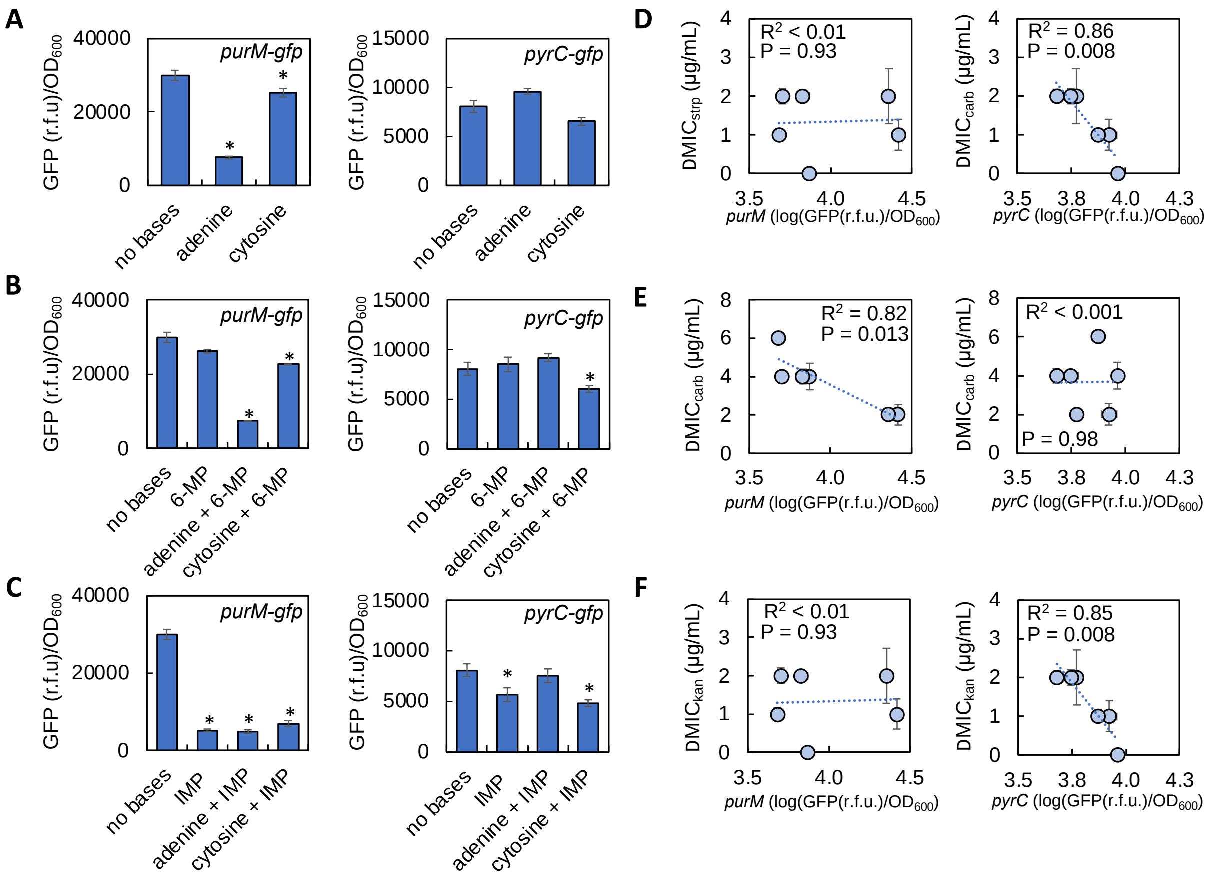

Fig. S18: Reporter activity after 24 hours of growth.

A) GFP (in relative fluorescent units, r.f.u.) of *purM*-gfp (left) and *pyrC*-gfp (right) in response to nitrogenous bases. * = different from no nitrogenous base control (P ≤ 0.045, two-tailed t-test). For all panels, GFP normalized by cell density (OD_{600}). Average from 5 biological replicates. Nitrogenous bases that were provided: 5 mM adenine and 10 mM cytosine. Error bars = SEM.

B) GFP of *purM*-gfp (left) and *pyrC*-gfp (right) in response to 0.05 µg/mL 6-MP and nitrogenous bases. * = different from no nitrogenous base control (P ≤ 0.041, two-tailed t-test).

C) GFP of *purM*-gfp (left) and *pyrC*-gfp (right) in response to 1 mM IMP and nitrogenous bases. * = different from no nitrogenous base control (P ≤ 0.030, two-tailed t-test).

D) Linear correlation between ΔMIC of streptomycin (strp) and GFP/OD_{600} from either *purM*-gfp (left) and *pyrC*-gfp (right) reporter strains. For panels D-F, P and R^2 values from linear regression and ΔMIC from Fig. 6. Weighted least squares (WLS) regression (*purM*: $R^2 < 0.01$, P = 0.90; *pyrC*: $R^2 = 0.86$, P = 0.024); Deming regression (*purM* - P = 0.93; *pyrC* P = 0.008).

E) Linear correlation between ΔMIC of carbenicillin (carb) and GFP/OD_{600} from either *purM*-gfp (left) and *pyrC*-gfp (right) reporter strains. ΔMIC from Fig. 6. WLS (*purM*: $R^2 = 0.79$, P = 0.017; *pyrC*: $R^2 = 0.27$, P = 0.29) and Deming regression (*purM* - P = 0.013; *pyrC* P = 0.98).

F) Linear correlation between ΔMIC of kanamycin (kan) and GFP/OD_{600} from either *purM*-gfp (left) and *pyrC*-gfp (right) reporter strains. ΔMIC from Fig. S19. WLS (*purM*: $R^2 < 0.01$, P = 0.96; *pyrC*: $R^2 = 0.84$, P = 0.028) and Deming regression (*purM* - P = 0.93; *pyrC* P = 0.008).

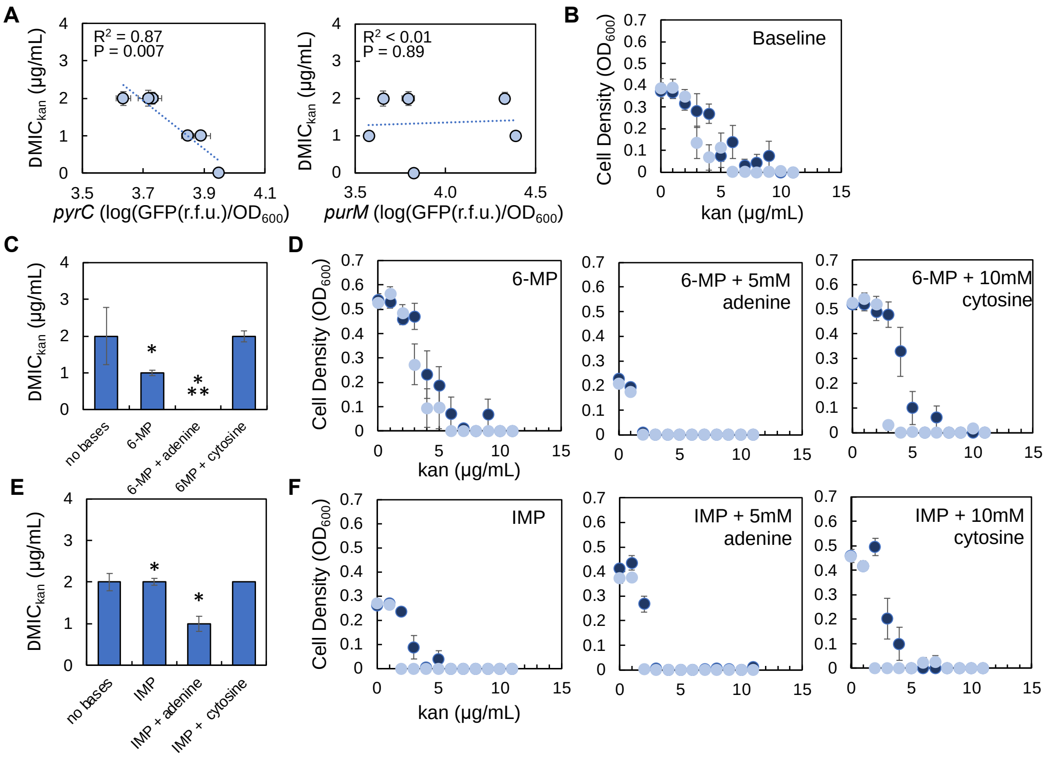

Fig. S19: Transcriptional activity of pyrimidine synthesis correlates with ΔMIC of the aminoglycoside kanamycin.

A) ΔMIC of kanamycin (kan) as a function of *pyrC* (left), and *purM* (right) reporter transcriptional activity. R^2 and P value from a linear regression. Weighted least squares (WLS) regression (*purM*: $R^2 < 0.01$, P = 0.99; *pyrC*: $R^2 = 0.87$, P = 0.02); Deming regression (*purM* - P = 0.89; *pyrC* P = 0.007). Error bars = SEM.

B) Raw data from which the baseline ΔMIC of kan was determined. Casamino acids = 0.1%. No nitrogenous bases are included. Dark blue = high density, light blue = low density. Error bars = SEM. Average from = 5 biological replicates.

C) ΔMIC of kan for *E. coli* grown in M9 medium supplemented with 6-MP, 5 mM adenine, and 10 mM cytosine. * = different than no nitrogenous base/no inhibitor control (P ≤ 0.02, two-tailed t-test). ** = not different than zero (P = 0.089, one-tailed t-test). SEM from 5 biological replicates. All P values in Table S10.

D) Raw data from which the baseline ΔMIC of kan was determined in the presence of 6-MP. Casamino acids = 0.1%. No nitrogenous bases are included. Dark blue = high density, light blue = low density. Error bars = SEM. Average from = 5 biological replicates.

E) ΔMIC of kan for *E. coli* grown in M9 medium supplemented with IMP, 5 mM adenine, and 10 mM cytosine. * = different than no nitrogenous base/no inhibitor control (P ≤ 0.003, two-tailed t-test). SEM from 6 biological replicates. All P values in Table S10.

F) Raw data from which the baseline ΔMIC of kan was determined in the presence of IMP. Casamino acids = 0.1%. No nitrogenous bases are included. Dark blue = high density, light blue = low density. Error bars = SEM. Average from = 6 biological replicates.

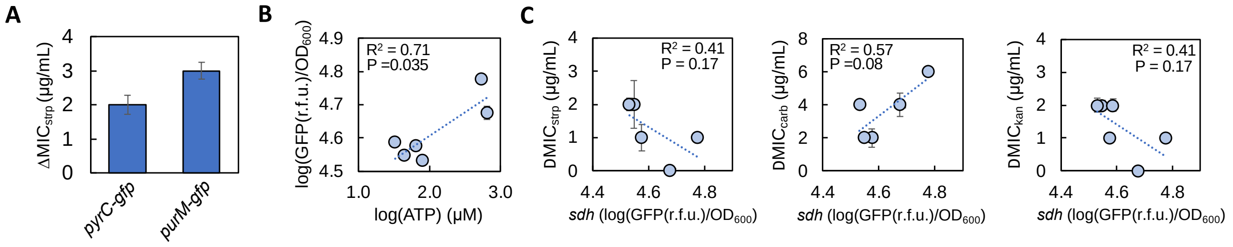

Fig. S20: Control experiments and succinate dehydrogenase (*sdh*) promoter activity as determined using reporter strains.

- **A)** ΔMIC of streptomycin (strp) for *purM-gfp* and *pyrC-gfp* reporter strains. Average from 6 biological replicates. Error bars = SEM.
- **B)** Linear correlation between [ATP] and *sdh*-gfp reporter activity. P and R^2 value from linear regression. [ATP] from Fig. 6.
- **C)** Linear correlation between GFP/OD_{600} of *sdh*-gfp reporter strain and ΔMIC of strp, kanamycin (kan), and carbenicillin (carb). ΔMIC for strep and carb from Fig. 3, ΔMIC for kan from Fig. S19.

Supplemental Tables

Table S1: Upper and lower bound flux values used for FBA simulations in Figs. 1, 5, 6, and 7. All other flux values were left unchanged in the model.

Reaction	Lower bound	Upper bound	Ref
**Core reactions**			
Glycine	-0.8835861	1000	(39)
Alanine	-1.682609333	1000	(39)
Arginine	-1.6722119	1000	(39)
Asparagine	-2.396529233	1000	(39)
Aspartate	0.895297803	1000	(39)
Cysteine	-1.4220841	1000	(39)
Glutamate	-1.5974812	1000	(39)
Glutamine	-1.987637733	1000	(39)
Histidine	-3.3186743	1000	(39)
Isoleucine	-2.090270167	1000	(39)
Leucine	-2.090270167	1000	(39)
Lysine	-2.177075267	1000	(39)
Methionine	-2.431328733	1000	(39)
Phenylalanine	-2.422301767	1000	(39)
Proline	-2.3791969	1000	(39)
Serine	-0.403842533	1000	(39)
Threonine	-1.907954967	1000	(39)
Tryptophan	-3.014569033	1000	(39)
Tyrosine	-3.524060967	1000	(39)
Valine	-2.266623633	1000	(39)
Thiamine	-0.000001	1000	(43)
Oxygen (O2tex)	-20	1000	(1, 42)
Glucose	-18	1000	(40, 41)
**Values specific to Fig. 6 and 7**			
6-MP (GLUPRT)	0	1.5	Supplemental results
IMP (Ex_IMP_e)	-1	1000	Supplemental results
**Values specific for Fig. 7**			
Adenine (Ex_ade_e)	-1	1000	Supplemental results
Cytosine (Ex_csn_e)	-0.5	1000	Supplemental results

Table S2: P values and the exact number of biological replicates (*n*) for Fig. 2. P value was determined using a two-tailed t-test against the no nitrogenous base control.

Condition	[nitrogenous base] mM	% CAA	*n* (growth rate)	P value (growth rate)	*n* ([ATP])	P value ([ATP])
**No nitrogenous bases**	0	0.01	7	1	4	1
		0.05	8			
		0.1	8			
		0.5	8			
		1	8			
**Adenine**	1	0.01	7	0.567		6.252E-11
		0.05	7			
		0.1	5			
		0.5	8			
		1	7			
	5	0.01	8	5.47E-16		2.17E-14
		0.05	8			
		0.1	6			
		0.5	6			
		1	7			
	10	0.01	7	2.30E-21		6.91E-11
		0.05	5			
		0.1	7			
		0.5	6			
		1	5			
**Cytosine**	1	0.01	7	2.75E-04		8.11E-04
		0.05	6			
		0.1	6			
		0.5	8			
		1	8			
	5	0.01	8	0.107		0.003
		0.05	7			
		0.1	7			
		0.5	6			
		1	8			
	10	0.01	7	0.437		0.244
		0.05	6			
		0.1	8			
		0.5	7			
		1	6			
**Thymine**	1	0.01	8	0.004		4.948E-04
		0.05	6			
		0.1	8			
		0.5	8			
		1	8			
	5	0.01	8	3.75E-13		1.78E-09

		0.05	8			
		0.1	8			
		0.5	7			
		1	7			
	10	0.01	8	1.101E-17		3.644E-11
		0.05	4			
		0.1	8			
		0.5	8			
		1	5			
**Uracil**	1	0.01	7	0.386		0.002
		0.05	9			
		0.1	10			
		0.5	10			
		1	12			
	5	0.01	9	0.276		0.002
		0.05	12			
		0.1	9			
		0.5	8			
		1	12			
	10	0.01	8	0.639		0.004
		0.05	7			
		0.1	7			
		0.5	11			
		1	12			

Table S3: Average residual values for growth curve fitting to determine growth rates in Fig. 2.

Growth condition	% CAA	Biological replicate												Average	SEM
no nitrogenous bases	**0.01**	0.22	0.09	0.04	0.02	0.05	0.09	0.06	-	-	-	-	-	0.09	0.02
	**0.05**	0.15	0.14	0.03	0.20	0.09	0.08	0.10	0.05	-	-	-	-	0.13	0.02
	**0.1**	0.16	0.20	0.26	0.18	0.11	0.12	0.15	0.14	-	-	-	-	0.20	0.02
	**0.5**	0.13	0.10	0.07	0.12	0.08	0.08	0.11	0.10	-	-	-	-	0.11	0.01
	**1**	0.07	0.07	0.15	0.09	0.07	0.04	0.05	0.05	-	-	-	-	0.09	0.01
1 mM adenine	**0.01**	0.03	0.04	0.18	0.04	0.06	0.14	0.25	-	-	-	-	-	0.07	0.03
	**0.05**	0.02	0.01	0.08	0.08	0.31	0.21	0.10	-	-	-	-	-	0.05	0.04
	**0.1**	0.11	0.13	0.18	0.11	0.45	-	-	-	-	-	-	-	0.13	0.06
	**0.5**	0.08	0.06	0.06	0.07	0.09	0.08	0.20	0.27	-	-	-	-	0.07	0.03
	**1**	0.08	0.04	0.06	0.09	0.13	0.11	0.08	-	-	-	-	-	0.07	0.01
5 mM adenine	**0.01**	0.33	0.11	0.09	0.08	0.02	0.03	0.18	0.10	-	-	-	-	0.15	0.03
	**0.05**	0.01	0.08	0.14	0.09	0.12	0.18	0.22	-	-	-	-	-	0.08	0.02
	**0.1**	0.33	0.08	0.06	0.05	0.05	0.02	-	-	-	-	-	-	0.13	0.04
	**0.5**	0.13	0.19	0.15	0.04	0.08	0.08	-	-	-	-	-	-	0.13	0.02
	**1**	0.16	0.17	0.05	0.08	0.06	0.08	0.05	-	-	-	-	-	0.11	0.02
10 mM adenine	**0.01**	0.02	0.07	0.19	0.05	0.16	0.11	0.12	-	-	-	-	-	0.08	0.02
	**0.05**	0.25	0.27	0.02	0.07	0.14	-	-	-	-	-	-	-	0.15	0.04
	**0.1**	0.15	0.15	0.02	0.12	0.08	0.18	0.01	-	-	-	-	-	0.11	0.02
	**0.5**	0.04	0.13	0.17	0.14	0.26	0.13	-	-	-	-	-	-	0.12	0.03
	**1**	0.16	0.01	0.10	0.04	0.05	-	-	-	-	-	-	-	0.08	0.02
1 mM cytosine	**0.01**	0.05	0.05	0.03	0.16	0.03	0.13	0.13	-	-	-	-	-	0.07	0.02
	**0.05**	0.07	0.07	0.04	0.28	0.25	0.17	-	-	-	-	-	-	0.11	0.04
	**0.1**	0.20	0.25	0.06	0.30	0.14	0.27	-	-	-	-	-	-	0.20	0.03
	**0.5**	0.08	0.11	0.07	0.10	0.07	0.09	0.15	0.10	-	-	-	-	0.09	0.01
	**1**	0.07	0.06	0.05	0.24	0.11	0.08	0.09	0.06	-	-	-	-	0.11	0.02
5 mM cytosine	**0.01**	0.05	0.09	0.04	0.05	0.02	0.03	0.12	0.14	-	-	-	-	0.06	0.01
	**0.05**	0.10	0.12	0.06	0.36	0.12	0.21	0.06	-	-	-	-	-	0.16	0.04
	**0.1**	0.09	0.08	0.22	0.08	0.15	0.22	0.13	-	-	-	-	-	0.12	0.02
	**0.5**	0.09	0.09	0.10	0.08	0.24	0.41	-	-	-	-	-	-	0.09	0.05
	**1**	0.03	0.15	0.11	0.35	0.13	0.23	0.16	0.08	-	-	-	-	0.16	0.03
10 mM cytosine	**0.01**	0.16	0.11	0.02	0.07	0.04	0.08	0.06	-	-	-	-	-	0.09	0.02
	**0.05**	0.25	0.09	0.11	0.08	0.22	0.12	-	-	-	-	-	-	0.13	0.03
	**0.1**	0.20	0.25	0.12	0.12	0.17	0.09	0.12	0.09	-	-	-	-	0.17	0.02
	**0.5**	0.21	0.12	0.32	0.12	0.09	0.12	0.09	-	-	-	-	-	0.19	0.03
	**1**	0.12	0.12	0.09	0.08	0.08	0.05	-	-	-	-	-	-	0.10	0.01
1 mM thymine	**0.01**	0.04	0.11	0.19	0.15	0.05	0.06	0.12	0.15	-	-	-	-	0.12	0.02
	**0.05**	0.12	0.25	0.12	0.23	0.17	0.11	-	-	-	-	-	-	0.18	0.02
	**0.1**	0.24	0.17	0.13	0.10	0.20	0.13	0.23	0.21	-	-	-	-	0.16	0.02

	**0.5**	0.11	0.21	0.10	0.12	0.07	0.23	0.16	0.10	-	-	-	-	0.14	0.02
	**1**	0.05	0.28	0.06	0.05	0.25	0.13	0.16	0.08	-	-	-	-	0.11	0.03
**5 mM thymine**	**0.01**	0.09	0.15	0.09	0.20	0.04	0.08	0.13	0.09	-	-	-	-	0.13	0.02
	**0.05**	0.23	0.32	0.14	0.11	0.14	0.01	0.02	0.13	-	-	-	-	0.20	0.03
	**0.1**	0.19	0.16	0.01	0.30	0.00	0.16	0.15	0.00	-	-	-	-	0.16	0.04
	**0.5**	0.09	0.14	0.11	0.27	0.12	0.18	0.09	-	-	-	-	-	0.15	0.02
	**1**	0.14	0.09	0.06	0.06	0.13	0.18	0.20	-	-	-	-	-	0.09	0.02
**10 mM thymine**	**0.01**	0.24	0.08	0.04	0.06	0.05	0.05	0.08	0.06	-	-	-	-	0.11	0.02
	**0.05**	0.13	0.10	0.15	0.09	-	-	-	-	-	-	-	-	0.12	0.01
	**0.1**	0.30	0.35	0.10	0.07	0.13	0.11	0.15	0.10	-	-	-	-	0.21	0.03
	**0.5**	0.25	0.21	0.12	0.07	0.07	0.14	0.21	0.21	-	-	-	-	0.16	0.02
	**1**	0.11	0.17	0.11	0.13	0.08	-	-	-	-	-	-	-	0.13	0.01
**1 mM uracil**	**0.01**	0.01	0.00	0.03	0.07	0.01	0.01	0.01	-	-	-	-	-	0.03	0.01
	**0.05**	0.05	0.09	0.06	0.25	0.22	0.10	0.03	0.03	0.36	-	-	-	0.11	0.04
	**0.1**	0.07	0.07	0.09	0.06	0.11	0.12	0.38	0.34	0.37	0.30	-	-	0.07	0.04
	**0.5**	0.08	0.10	0.33	0.05	0.21	0.17	0.11	0.05	0.07	0.07	-	-	0.14	0.03
	**1**	0.06	0.11	0.11	0.09	0.12	0.10	0.16	0.14	0.04	0.05	0.05	0.04	0.09	0.01
**5 mM uracil**	**0.01**	0.03	0.47	0.04	0.01	0.14	0.00	0.03	0.01	0.01	-	-	-	0.14	0.05
	**0.05**	0.04	0.26	0.17	0.35	0.18	0.13	0.21	0.12	0.09	0.25	0.32	0.35	0.21	0.03
	**0.1**	0.27	0.14	0.08	0.08	0.09	0.24	0.24	0.20	0.24	-	-	-	0.14	0.02
	**0.5**	0.06	0.13	0.12	0.10	0.10	0.07	0.07	0.07	-	-	-	-	0.10	0.01
	**1**	0.07	0.07	0.09	0.07	0.14	0.07	0.08	0.08	0.05	0.04	0.05	0.05	0.07	0.01
**10 mM uracil**	**0.01**	0.04	0.05	0.02	0.15	0.01	0.06	0.04	0.13	-	-	-	-	0.06	0.02
	**0.05**	0.04	0.13	0.29	0.05	0.04	0.37	0.05	-	-	-	-	-	0.13	0.05
	**0.1**	0.04	0.36	0.17	0.20	0.08	0.06	0.41	-	-	-	-	-	0.19	0.05
	**0.5**	0.05	0.09	0.11	0.13	0.31	0.15	0.36	0.10	0.08	0.09	0.09	-	0.10	0.03
	**1**	0.13	0.07	0.07	0.10	0.07	0.05	0.05	0.05	0.06	0.04	0.05	0.05	0.09	0.01

Table S4: Average residual values for growth curve fitting to determine growth rates for *P. aeruginosa* in Fig. 4.

Growth condition	% CAA	Biological replicate						Average	SEM
no nitrogenous bases	**0.1**	0.26	0.15	0.33	0.41	0.15	-	0.26	0.04
	**0.5**	0.27	0.32	0.49	0.22	0.25	0.46	0.34	0.04
	**1**	0.17	0.22	0.45	0.11	0.27	0.17	0.23	0.04
5 mM adenine	**0.1**	0.27	0.16	0.00	0.17	0.29	-	0.18	0.05
	**0.5**	0.16	0.24	0.42	0.15	0.30	0.16	0.24	0.04
	**1**	0.22	0.23	0.41	0.22	0.36	0.32	0.29	0.03
10 mM cytosine	**0.1**	0.18	0.27	0.22	0.27	0.19	-	0.23	0.02
	**0.5**	0.20	0.35	0.13	0.19	0.21	-	0.21	0.03
	**1**	0.41	0.28	0.21	0.31	0.21	-	0.28	0.03
10 mM thymine	**0.1**	0.15	0.13	0.22	0.25	0.23	-	0.19	0.02
	**0.5**	0.12	0.37	0.17	0.12	-	-	0.20	0.05
	**1**	0.21	0.29	0.43	0.20	0.30	0.40	0.31	0.04
10 mM uracil	**0.1**	0.16	0.21	0.20	0.45	0.23	-	0.25	0.05
	**0.5**	0.11	0.13	0.33	0.20	0.19	0.19	0.19	0.03
	**1**	0.14	0.38	0.10	0.24	0.42	-	0.26	0.06

Table S5: P values and the exact number of biological replicates (*n*) for Fig. 4. P values for growth rate and [ATP] were determined using a two-tailed t-test. P values for ATP and growth rate from two-tailed t-test. ΔMIC from a one-tailed t-test relative to nitrogenous base control. We used a one-tailed t-test as we only asked if ΔMIC could decrease (but not increase); thus, we asked our question in one direction only. strp = streptomycin; carb = carbenicillin.

Condition	[nitrogenous base] mM	% CAA	*n* (growth rate)	P value (growth rate)	*n* ([ATP])	P value ([ATP])	P value (ΔMIC strp)	P value (ΔMIC carb)
No nitrogenous bases (control)	0	0.1	5	1.000	3	1.000	0.5	0.5
		0.5	6		3			
		1	6		3			
Adenine	5	0.1	5	0.069	4	0.032	0.385	0.051
		0.5	6		4			
		1	6		4			
Cytosine	10	0.1	5	0.205	4	0.446	0.067	0.10
		0.5	5		4			
		1	5		4			
Thymine	10	0.1	5	0.231	4	0.669	0.041	0.037
		0.5	4		4			
		1	6		4			
Uracil	10	0.1	5	0.520	4	0.130	0.283	0.28
		0.5	6		4			
		1	5		4			

Table S6: P values and exact number of biological replicates (*n*) for knockout strains in Fig. 5. P values were determined using a two-tailed t-test and compared to wildtype strain.

Strain	[equimolar nitrogenous bases] µM	*n* (growth rate)	P value (growth rate)	*n* ([ATP])	P value ([ATP])	P value (ΔMIC - strp)	P value (MIC - carb)
wildtype (*wt*)	1	7	1	3	1	1	1
	4	16		3			
	7	13		3			
ΔpyrC	1	13	< 0.0001	3	<0.001	0.038	0.025
	4	6		3			
	7	5		3			
ΔpurK	1	19	< 0.0001	3	0.010	0.184	0.004
	4	11		3			
	7	10		3			

Table S7: Average residual values for growth curve fitting to determine growth rates for knockout strains. *wt* = wildtype.

Strain	Base	Biological replicate																				Average Residual	SEM	Overall Average	Overall SEM
	1	0.09	0.15	0.16	0.15	0.17	0.15	0.22														0.16	0.01		
	4	0.30	0.16	0.18	0.16	0.15	0.14	0.11	0.10	0.17	0.19	0.17	0.15	0.16	0.16	0.17	0.31					0.17	0.01		
wt	7	0.18	0.12	0.38	0.19	0.39	0.20	0.07	0.09	0.42	0.50	0.31	0.16	0.13								0.24	0.04	0.19	0.03
	1	0.03	0.19	0.25	0.06	0.05	0.06	0.06	0.16	0.21	0.27	0.24	0.11	0.18								0.14	0.02		
	4	0.23	0.06	0.16	0.25	0.40	0.30															0.24	0.04		
Δ*pyrC*	7	1.31	1.97	1.94	1.59	1.55																1.67	0.11	0.49	0.10
	1	0.12	0.11	0.10	0.24	0.25	0.13	0.15	0.13	0.10	0.10	0.07	0.07	0.05	0.05	0.12	0.01	0.07	0.07	0.06	0.07	0.10	0.01		
	4	0.45	0.01	0.27	0.37	0.12	0.11	0.03	0.03	0.22	0.11	0.14										0.17	0.04		
Δ*purK*	7	0.34	0.18	0.26	0.28	0.39	0.48	0.42	0.27	0.43	0.31											0.34	0.03	0.18	0.03

Table S8: Average residual values for growth curve fitting to determine growth rates in Fig. 6.

Growth condition	Nitrogenous base	Biological replicate						Average	SEM
1mM IMP	No bases	0.02	0.01	0.17	0.02	0.08	-	0.06	0.03
	5 mM adenine	0.15	0.11	0.10	0.07	0.12	0.04	0.10	0.01
	10 mM cytosine	0.12	0.14	0.25	0.19	0.16	-	0.17	0.02
0.05 µg/mL 6-MP	No bases	0.10	0.15	0.15	0.14	0.17	0.17	0.15	0.01
	5 mM adenine	0.06	0.13	0.07	0.06	0.05	0.01	0.07	0.01
	10 mM cytosine	0.10	0.12	0.09	0.21	0.17	0.10	0.13	0.02

Table S9: P values and the exact number of biological replicates (*n*) for experiments in Fig. 6. P values were determined using a two-tailed t-test and compared to wildtype strain and without inhibitor and nitrogenous base supplementation.

Growth condition	Nitrogenous base	Biological replicates (*n*) for growth rate	P values for growth rate	Biological replicates (*n*) for [ATP]	P values for [ATP]
1 mM IMP	No bases	5	0.026	6	0.078
	5 mM adenine	6	< 0.0001	6	< 0.0001
	10 mM cytosine	5	< 0.0001	6	0.0007
0.05 µg/mL 6-MP	No bases	6	0.32	6	0.031
	5 mM adenine	6	< 0.0001	6	< 0.0001
	10 mM cytosine	6	0.923	6	0.002

Table S10: P values and the exact number of biological replicates (*n*) for MIC experiments with kanamycin. P values were determined using a two-tailed t-test and compared to wildtype strain and without inhibitor/nitrogenous base supplementation.

Inhibitor	Nitrogenous base	Biological replicates (*n*) for growth rate	P value (ΔMIC)
none	none	5	1
6-MP	none	5	0.02
	5 mM adenine	5	<0.001
	10 mM cytosine	6	0.876
IMP	none	6	0.001
	5 mM adenine	6	0.003
	10 mM cytosine	6	0.184
